# Supplementary material for: Acquisition of a Quantitative, Stoichiometrically Conserved Ratiometric Marker of Maturation Status in Stem Cell-Derived Cardiac Myocytes
Source: Stem Cell Reports. 2014 Sep 4;3(4):594–605. doi: 10.1016/j.stemcr.2014.07.012 (PMC4223713; doi:10.1016/j.stemcr.2014.07.012)
Supplement: Document S1. Supplemental Experimental Procedures and Figures S1–S5 [file mmc1.pdf]

Stem Cell Reports, Volume *3*

Supplemental Information

Acquisition of a Quantitative, Stoichiometrically Conserved Ratiometric Marker of Maturation Status in Stem Cell-Derived Cardiac Myocytes

Fikru B. Bedada, Sunny S-K. Chan, Stefania K. Metzger, Liyang Zhang, Jianyi Zhang, Daniel J. Garry, Timothy J. Kamp, Michael Kyba, and Joseph M. Metzger

Figure S1 related to Figure 1 and 2

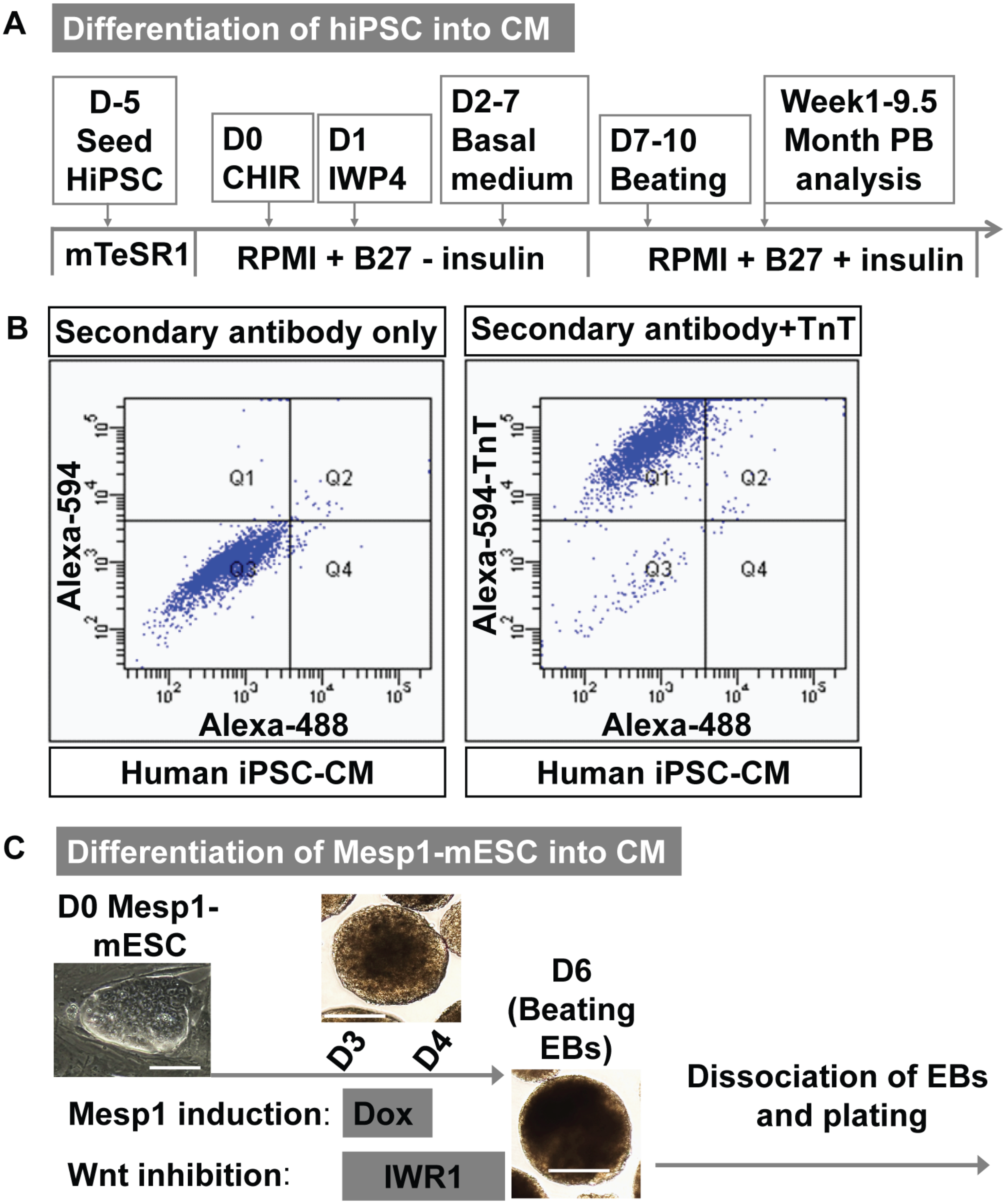

Figure S2 related to figure 1

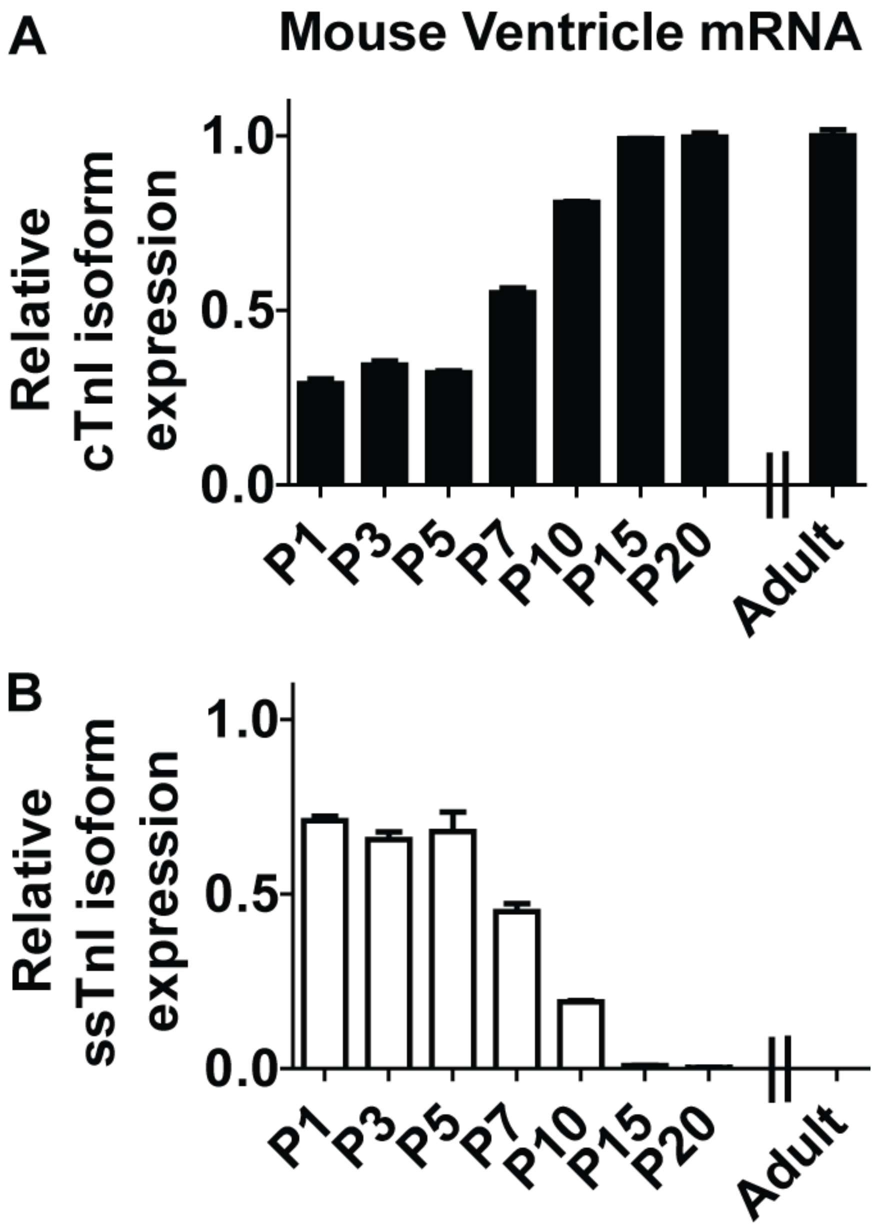

Figure S3 related to figure 1 and 2

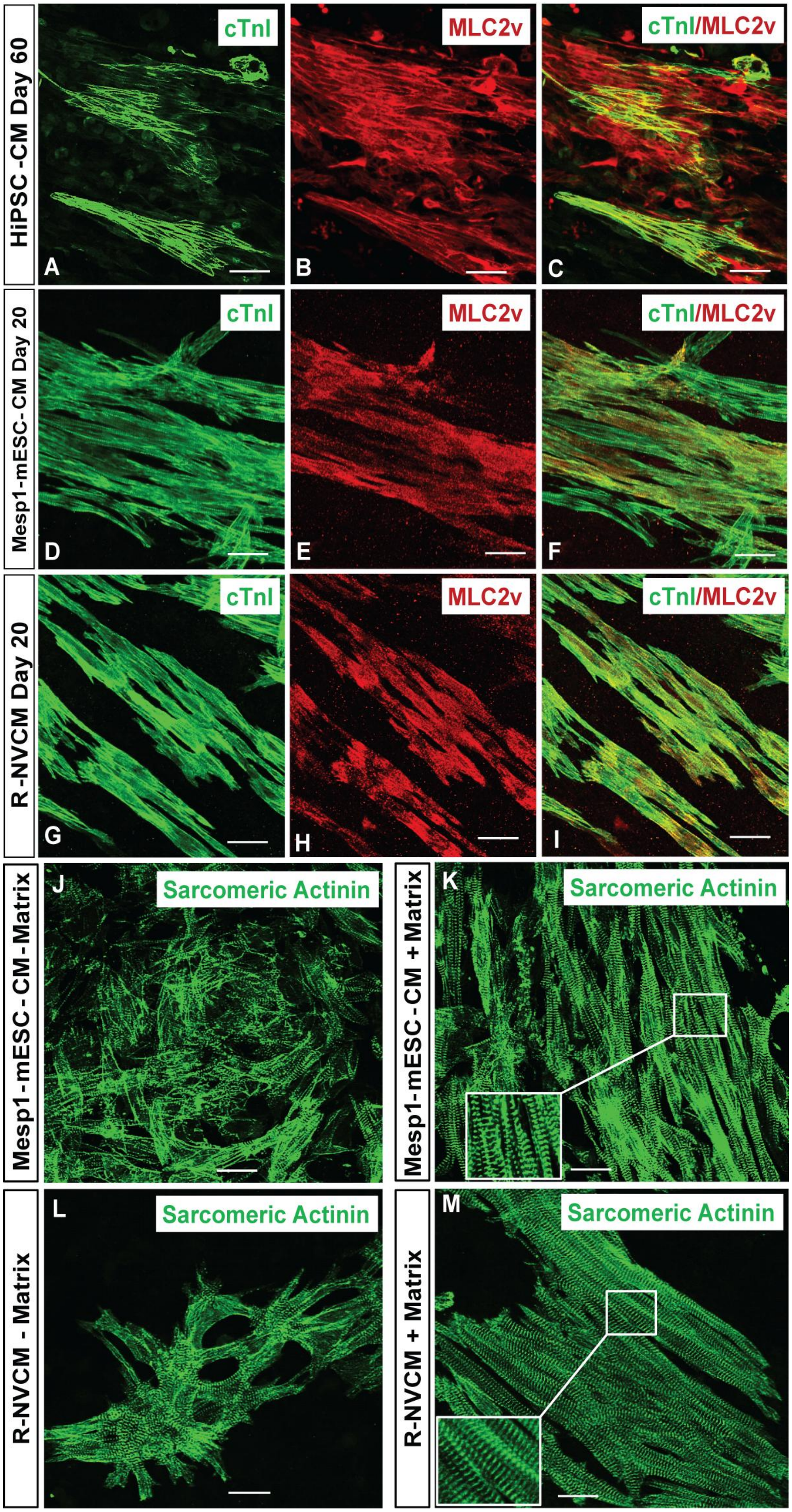

Figure S4 related to figure 2

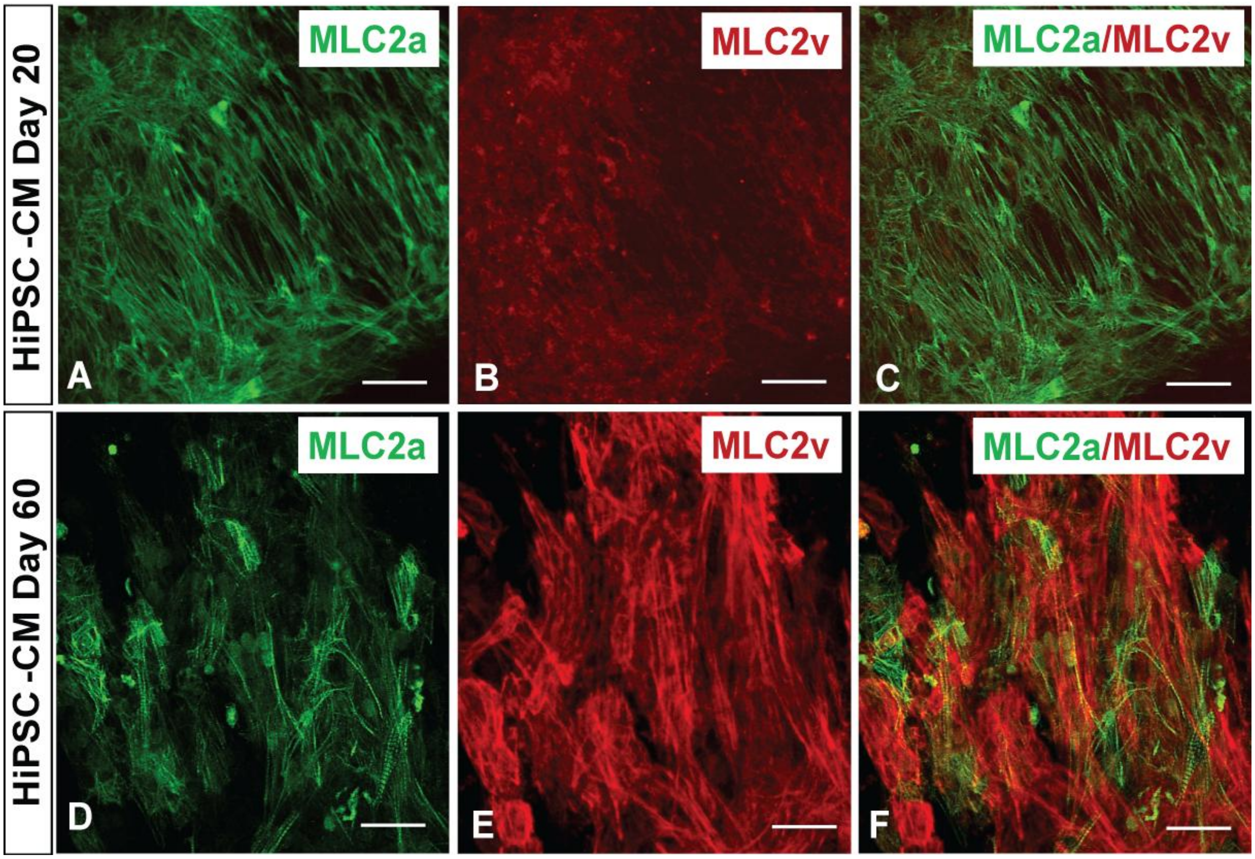

Figure S5 related to figure 5

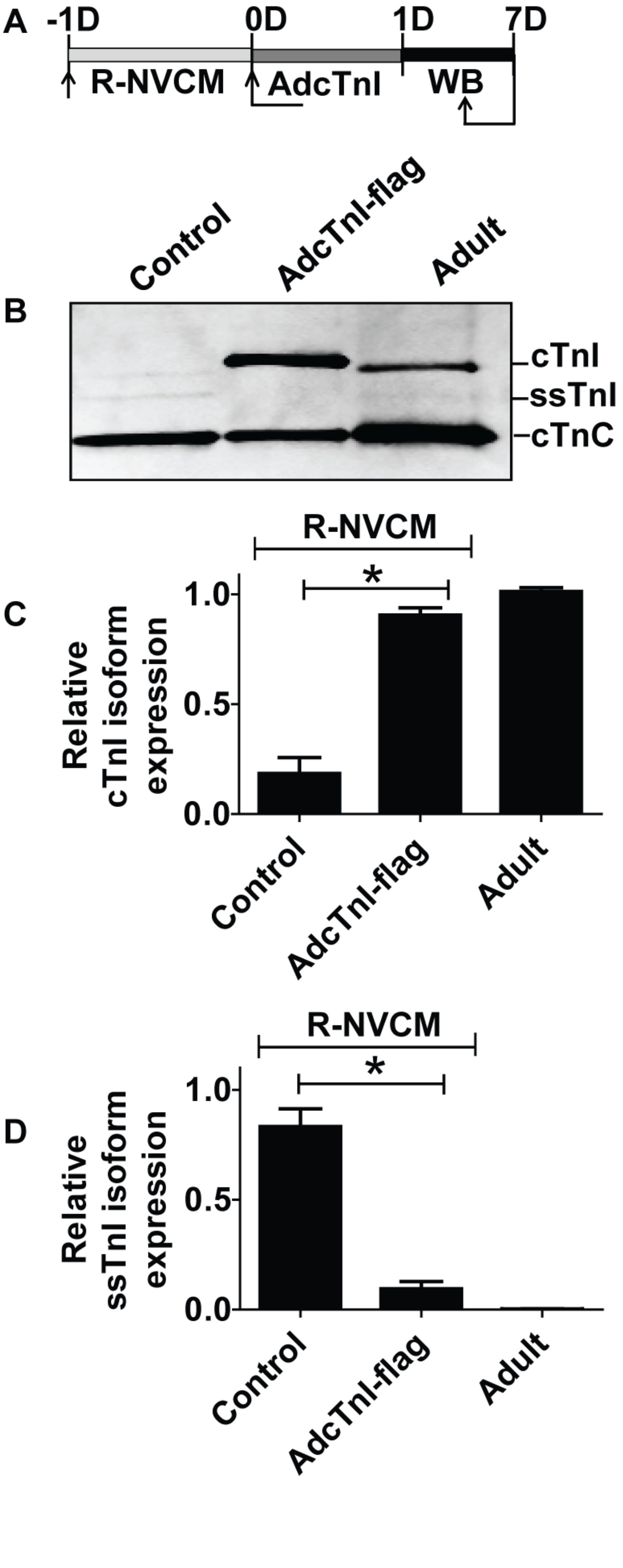

Supplemental Figure Legends

Figure S1 related to figure 1 and 2. Differentiation protocol for hiPSC and Mesp1-mESC-CMs. (A) Schematic of the differentiation protocol for hiPSC-CMs as described in the material and methods. (B) FACS analysis of TnT expression and (C) schematic of differentiation protocol for Mesp1-mESC-CMs as described in the material and methods. The scale bar is 50 µm for undifferentiated mESC and 200 µm for day 4 and day 6 EBs.

Figure S2 related to figure 1. **Developmental expression of *TNNI* isoform mRNAs in ventricles of mouse pups in vivo.** The temporal developmental transition of ssTnI to cTnI was analyzed at the mRNA level to evaluate the transcriptional regulation in vivo. To this effect, RNA samples were obtained from ventricles of postnatal mouse pups at different time points including P1-P20. Ventricles from an adult mouse used as a control. Quantitative RT-PCR data shows gradual increase in the level of cTnI and concomitant reduction in ssTnI for mouse pups in vivo. Data analyzed by two-way ANOVA with Bonferroni post-hoc test and expressed as mean ± SEM, n=3 independent experiments, $P < 0.05$.

Figure S3 related to figure 1 and 2. Sarcomeric alignment and co-localization of mature and chamber specific markers in hiPSC-CMs and rodent-CMs. Immunohistochemistry was used to evaluate the localization of cTnI (mature marker) and MLC2v (ventricular marker) in 60 days post spontaneous beating hiPSC-CM (top panels A-C); Mesp1-mESC-CMs (middle panels D-F) and R-NVCMs (lower panels G-I). The images were taken with 40x objective; calibration bar is 50 µm. Rodent-CMs repopulating the biological matrix aligned and displayed distinct sarcomere organization within 7-15 days of culture with extensive synchronized spontaneous contraction compared with those cultured in the absence of biological matrix. Mesp1-mESC-CM cultured in the absence of the biological matrix did not align well in culture (Panel J left) whereas Mesp1-mESC-CM repopulating the biological matrix aligned and displayed marked sarcomere organization (Panel K right). R-NVCM cultured in the absence of the biological matrix did not align well (Panel L left) whereas R-NVCM repopulating the biological matrix aligned and displayed marked sarcomere organization (Panel M right). The images were taken with 40x objective; calibration bar is 50 µm.

Figure S4 related to figure 2. Acquisition of chamber specific markers in hiPSC-CMs. HiPSC-CMs were stained with MLC2a (green) and MLC2v (red) specific antibodies. Representative images are shown for day 20 MLC2a (A), MLC2v (B) and merged images (C). Day 60 MLC2a is shown in (D), MLC2v (E) and merged images (F). The images were taken with 40x objective; calibration bar is 50 μm.

Figure S5 related to figure 5. Acquisition of the mature signature by stoichiometric gene replacement in rodent-CM. Schematic of AdcTnI (flag tagged) gene transduction in day 1 R-NVCM (A). Western blot of AdcTnI(flag tagged) treated R-NVCM is shown in (B). The direct effect of AdcTnI(flag tagged) on acquisition of cTnI is quantified in (C) and suppression of ssTnI is quantified in (D).). cTnC is used as cardiac specific loading control and for normalization/quantification. Data analyzed by One-way ANOVA with Bonferroni post-hoc test and expressed as mean ± SEM, n=3 independent experiments, ∗=P <0.05.

Supplemental Experimental Procedure

Preparation of thin slices of biological matrix for culture of hiPSC-CM and rodent-CM

Whole hearts were isolated from female Sprague–Dawley rats following anesthetization by isoflurane inhalation. Freshly isolated rat hearts were placed in 1 mm stainless steel rat heart slicer block with slice intervals spaced 1.0mm apart (Zivic Instruments). The 1.0 mm transverse section slice intervals allow slices of repeatable and reproducible uniformly sized sections. The 1 mm thin heart sections were placed in 1% SDS with continuous agitation and regular change of the detergent for 72 hrs. The matrix was washed and rinsed continuously with PBS for 2-3 days and kept at 4°c in PBS until usage.

Next, $18mm^2$ coverslips were placed in 6 well plates and the surface aseptically coated with 0.1mg/ml poly-D-lysine (Sigma) for 5 min and then rinsed with tissue culture grade water and allowed to dry for 2hrs. The thin slice of decellularized matrix was placed on poly-D-lysine-coated coverslips for 15-30min. Following this, hiPSC-CMs or rodent-CMs were placed in the decellularized cardiac thin sections for 1-2 hrs and medium changed every 2-3 days thereafter. As a control, hiPSC-CM or rodent-CM are cultured in coverslips coated with matrigel only.

Flow Cytometry Analysis

Dissociated single hiPSC-CMs were fixed with 2% (vol/vol) paraformaldehyde for 30min at room temperature and stained with TnT primary antibody for 1hr at room temperature and Alexa 594 secondary antibodies for 1hr at room temperature in PBS plus 0.01% (vol/vol) Triton X-100 and 1% (wt/vol) BSA. HiPSC-CMs that were stained with secondary antibody only were used as control for FACS gating. The expression was analyzed using BD FACSAriaII (BD Biosciences, San Diego, CA, USA).

Quantitative RT-PCR

Total RNA was isolated from hiPSC-CM, rodents-CMs using Trizol reagent (Invitrogen) following manufacturer's instructions. In the reverse transcription (RT) step, cDNA was reverse transcribed from 500 ng total RNA samples using TaqMan Reverse Transcription Kit. PCR products were amplified from cDNA samples using the SYBR Green PCR Master Mix (Invitrogen). All qPCR assays were performed in duplicate on all control and experimental group and normalized against control myocytes. Relative quantitation analysis of gene expression was conducted according to the $2^{-\Delta\Delta CT}$ method as described [1]. cTnC was used as endogenous internal standard for cTnI:ssTnI expression analysis to determine the abundance of amplified target gene within the same sample.

Supplemental References

1. Livak, K.J. and T.D. Schmittgen, *Analysis of relative gene expression data using real-time quantitative PCR and the 2(-Delta Delta C(T)) Method.* Methods, 2001. **25**(4): p. 402-8.
