# Supplementary material for: Acquisition of a Quantitative, Stoichiometrically Conserved Ratiometric Marker of Maturation Status in Stem Cell-Derived Cardiac Myocytes
Source: Stem Cell Reports. 2014 Sep 4;3(4):594–605. doi: 10.1016/j.stemcr.2014.07.012 (PMC4223713; doi:10.1016/j.stemcr.2014.07.012)
Supplement: Document S2. Article plus Supplemental Information [file mmc2.pdf]

Stem Cell Reports
Article
ISSCR
OPEN ACCESS

Acquisition of a Quantitative, Stoichiometrically Conserved Ratiometric Marker of Maturation Status in Stem Cell-Derived Cardiac Myocytes

Fikru B. Bedada,[1] Sunny S-K. Chan,[2] Stefania K. Metzger,[1] Liying Zhang,[2] Jianyi Zhang,[2] Daniel J. Garry,[2] Timothy J. Kamp,[3] Michael Kyba,[2] and Joseph M. Metzger[1,2,*]
[1]Department of Integrative Biology and Physiology, University of Minnesota Medical School, Minneapolis, MN 55455, USA
[2]Lillehei Heart Institute, University of Minnesota Medical School, Minneapolis, MN 55455, USA
[3]Department of Medicine, Stem Cell and Regenerative Medicine Center, University of Wisconsin, Madison, WI 53705, USA
*Correspondence: metzgerj@umn.edu
http://dx.doi.org/10.1016/j.stemcr.2014.07.012
This is an open access article under the CC BY license (http://creativecommons.org/licenses/by/3.0/).

SUMMARY

There is no consensus in the stem cell field as to what constitutes the mature cardiac myocyte. Thus, helping formalize a molecular signature for cardiac myocyte maturation would advance the field. In the mammalian heart, inactivation of the "fetal" *TNNI* gene, *TNNI1* (ssTnI), together in temporal concert with its stoichiometric replacement by the adult *TNNI* gene product, *TNNI3* (cTnI), represents a quantifiable ratiometric maturation signature. We examined the *TNNI* isoform transition in human induced pluripotent stem cell (iPSC) cardiac myocytes (hiPSC-CMs) and found the fetal *TNNI* signature, even during long-term culture. Rodent stem cell-derived and primary myocytes, however, transitioned to the adult TnI profile. Acute genetic engineering of hiPSC-CMs enabled a rapid conversion toward the mature TnI profile. While there is no single marker to denote the mature cardiac myocyte, we propose that tracking the cTnI:ssTnI protein isoform ratio provides a valuable maturation signature to quantify myocyte maturation status across laboratories.

INTRODUCTION

Cardiac myocytes derived from progenitors or from somatic cell reprogramming are attractive platforms for disease-modeling studies and have potential in regenerative medicine. One of the major challenges hindering full realization of stem cell-derived myocytes in the lab and clinic relates to uncertainties in the maturation status of the myocytes (Ieda et al., 2010; Hansson and Chien, 2012; Qian et al., 2012; Shiba et al., 2012). The well-characterized cardiac myocyte developmental profile in vivo can become disordered when myocytes are isolated and cultured in vitro. This developmental conundrum presents major challenges to tracking and documenting the maturation of stem cell-derived cardiac myocytes. Tracking the maturation status of human induced pluripotent stem cell-derived cardiac myocytes (hiPSC-CMs), for example, would be indispensable in ultimately using these cells as a platform for in vitro drug discovery or for potential therapeutic applications in vivo (Hansson and Chien, 2012; Palpant and Murry, 2012; Shiba et al., 2012).

To date, there is no clear agreement in the stem cell field as to what constitutes the mature cardiac myocyte or which specific marker, if any, can accurately track the differentiation status of stem cell-derived myocytes. For instance, when primary cardiac myocytes are isolated and cultured in vitro, they can re-express a myriad of embryonic/fetal protein isoforms. Further, other well-used structural or physiological markers, including Ca^{2+} handling, cell morphology/striation pattern, and beating, are also limited by developmental reversion under stress conditions and thus difficult to quantify as strict maturation markers. Collectively, these issues present challenges to investigators wishing to track the differentiation status of stem cell-derived cardiac myocytes. In this light, implementation of a quantifiable molecular signature for tracking the differentiation status of cardiac myocytes would advance the field.

We propose that a quantitative marker for tracking cardiac myocyte maturation resides in the developmentally controlled and irreversible (excepting targeted reprogramming) genetic switch in the sarcomeric *TNNI* gene. Owing to strict developmental control in all mammalian hearts, including humans, the programmed inactivation of the "fetal" *TNNI* gene, *TNNI1*, together in exquisite temporal concert with stoichiometric replacement by the adult gene, *TNNI3*, represents a unique molecular signature of the mature adult cardiac myocyte in vivo (Bhavsar et al., 1991; Sasse et al., 1993; Siedner et al., 2003). The troponin complex is composed of three subunits—troponin T (TnT), the tropomyosin binding subunit; troponin C (TnC), the calcium binding subunit; and troponin I (TnI), an inhibitory subunit (Dhoot et al., 1978; Ohtsuki et al., 1986; Parmacek and Solaro, 2004)—and controls the interaction of thick and thin filaments in response to alterations in intracellular Ca^{2+} concentrations (Ebashi, 1983, 1988; Lehman et al., 2001; Metzger and Westfall, 2004). The TnT subunit has been used extensively in attempt to identify the cardiac lineage (Chan et al., 2013b; Hazeltine et al., 2013; Kawamura et al., 2013; Park et al., 2014; Rebuzzini et al., 2013). However, cTnT is also expressed in noncardiac cells such as smooth muscle cells (Porrello et al., 2011, 2013; Lu

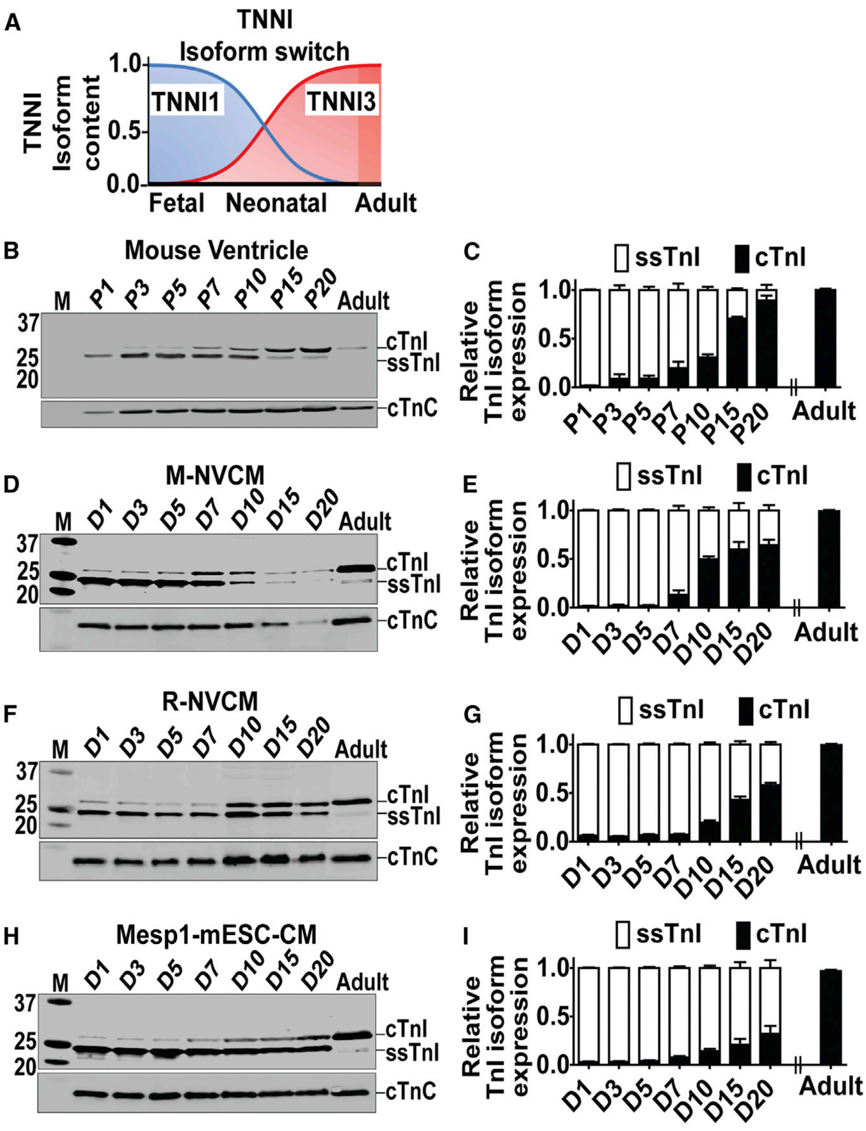

Figure 1. Temporal Expression Pattern of *TNNI* Protein Isoform Content in Rodent CMs

(A) Schematic of *TNNI* isoform switching time course during development of the mammalian heart.

(B and C) Western blot (B) and summary bar graph (C) of ventricles of mouse pups at postnatal days P1–P20. Ventricle from adult mouse is used as a control.

(D and E) Western blot (D) and summary bar graph (E) for TnI isoform content in M-NVCM cultured in vitro for D1–D20.

(F and G) Western blot (F) and summary bar graph (G) for R-NVCM cultured in vitro for D1–D20.

(H and I) Western blot (H) and summary bar graph (I) for Mesp1-mESC-CMs cultured in vitro for D1–D20.

See also Figures S1–S3. Data analyzed by two-way ANOVA with Bonferroni post hoc test and expressed as mean ± SEM, n = 3 independent experiments, $p < 0.05$ for (C), (E), (G), and (I).

et al., 2013; Lundy et al., 2013; Mahmoud et al., 2013; Xin et al., 2013), limiting its use as a cardiac lineage marker. Many other structural, regulatory, morphological, and metabolic markers have been used for cardiac lineage and maturation assignment; however, these are subject to reversion to the fetal program in stress and disease. For example, the MyH6 gene is highly cardiac specific, but a transition in myosin isoform content in disease complicates its use for lineage or maturation assignment (Sasse et al., 1993; Nakao et al., 1997; Reiser et al., 2001)

There are two isoforms of TnI in heart muscle, encoded on two separate genes that are expressed under strict developmental control in the mammalian heart (Hunkeler et al., 1991; MacGeoch et al., 1991; Metzger and Westfall, 2004). The *TNNI1* gene (slow skeletal TnI/ssTnI/cardiac fetal) is expressed in the sarcomeres of the fetal heart and in late fetal/early neonatal life and then fully extinguished such that there is no ssTnI protein detected in the adult myocardium (Bhavsar et al., 1991; Sasse et al., 1993; Siedner et al., 2003) (Figure 1A). The *TNNI3* gene (adult cardiac TnI/cTnI) is activated in fetal/early neonatal life and then is exclusively expressed in the adult myocardium. This strict 1:1 conversion is ideal for a differentiation status marker owing to tight preservation of sarcomere stoichiometry throughout development. This differs, for example, from tracking a marker that increases in maturation because the critical internal control of a reference "fetal" marker decreasing in parallel is lacking. Further, the ssTnI (fetal) to cTnI (adult) isoform stoichiometric conversion does not revert to the "fetal" gene program in cardiac stress or

disease (Sasse et al., 1993; Averyhart-Fullard et al., 1994; Huang et al., 2000), in distinction with reversible differentiation status markers currently in use (Razeghi et al., 2001; Lundy et al., 2013). The combined properties of stoichiometric conservation and general irreversibility are unique in combination and make the cTnI: ssTnI protein isoform ratio potentially highly useful as a quantitative marker of maturation status.

Using this marker of maturation, our findings show that rodent neonatal and murine embryonic stem cell (ESC)-derived cardiac myocytes in culture track the transition in the ssTnI to cTnI protein isoform switch comparable to the temporal pattern in vivo. In comparison, hiPSC-CMs beating continuously in culture retain a fetal-like state in terms of the TnI isoform profile regardless of differentiation condition, hiPSC line (five different hiPSC-CMs lines tested), and duration of culture (9+ months). By instituting a maturation status marker, as proposed here, myocytes derived from any progenitor/reprogramming approach and with any differentiation protocol can be directly and quantitatively compared across laboratories.

While the TnI isoform ratio is proposed here to be necessary for tracking maturation of stem cell-derived cardiac myocyte, it is not sufficient, on its own, to establish the mature adult cardiac myocyte state. Thus, we suggest that the TnI protein isoform profile be instituted as one indispensible component to be added to the myriad of maturation markers, including functional, morphological, metabolic and structural markers, required for the assignment of the mature adult cardiac myocyte state.

RESULTS

Temporal Pattern of ssTnI to cTnI Isoform Switching in Rodent-CMs In Vivo and In Vitro

To validate the TnI molecular signature of maturation in CMs, first we assessed the natural temporal developmental transition of ssTnI to cTnI in rodents in vivo. We isolated protein and RNA from ventricles of postnatal mouse pups at specific postnatal time points (days), postnatal day 1 (P1) to P20. Western blots determined greater than 90% cTnI expression with concomitant stoichiometrically conserved reduction of ssTnI protein in vivo at day 20 (Figures 1B and 1C). We observed a similar developmental transition pattern at the mRNA level (Figures S2A and S2B available online). Following the validation of TnI isoform maturation profile in vivo, we set out to assess the *TNNI* developmental expression pattern in vitro. Protein samples were collected at time points 1, 3, 5, 7, 10, 15, and 20 days after isolation and plating in culture and were analyzed for ssTnI and cTnI content. Western blots showed cTnI expression in cultured mouse neonatal cardiac myocytes that increased over time and with ssTnI expression stoichiometrically reduced in temporal concert with the increased cTnI expression (Figures 1D and 1E). This same general pattern was obtained in rat neonatal cardiac myocytes and in mouse Mesp-1 ESC-derived CMs (Figures 1F–1I). We also performed immunohistochemistry and found appropriate sarcomeric localization of cTnI in the rodent-CMs (Figures S3D and S3G). Additionally, in each case (neonatal or Mesp-1), CMs in the biological matrix aligned and displayed enhanced sarcomere organization (Figures S3J–S3M). However, refined sarcomeric organization and myocyte alignment did not affect the timing of the ssTnI:cTnI isoform conversion (Figures S3D and S3G).

Temporal Pattern of ssTnI to cTnI Switch in hiPSC-CMs

To gain insight into the molecular signature of cardiac myocyte maturation, hiPSC-CMs were studied in biological cardiac matrix with continuous spontaneous beating from 1 week to 9.5 months in vitro (Figure 2). To analyze the *TNNI* isoform expression pattern, protein samples were collected at time points 1, 2, 3, 4, 5, 6, and 7 weeks and 2, 6, and 9.5 months and ssTnI to cTnI (*TNNI* isoforms) content determined. Figure 2 documents the pattern of transition from *TNNI1* (ssTnI) to TNNI3 (cTnI) by western blot. Protein samples from adult human hearts were used as a positive control. Western blot data showed that ssTnI isoform content was dominant with no evidence of significant diminution even after culture for 9.5 months (Figures 2A and 2B). The hiPSC-CMs did express limited yet detectable amount of cTnI after 2 months of culture; however, this level did not change even up to 9.5 months of culture, the longest period studied. Western blot-based quantification showed only 2% of total TnI content was the cTnI isoform at the end of the study period (9.5 months of culture) and is evidence of stalled developmental maturation. In these experiments, to address issues of noncardiac growth during long-term culture, we used the RPMI and B27+ insulin cocktail that does not promote fibroblast outgrowth compared with culturing in fetal bovine serum (FBS).

Immunofluorescence (IF) showed sarcomere detection of cTnI in hiPSC-CMs (Figure S3A); however, when compared with the western blot data, this finding underscores the challenges of using IF to quantify this maturation marker. Structurally, as found in neonatal rodent and Mesp1-mESC derived CMs, hiPSC-CMs cultured on biological matrix aligned and displayed enhanced sarcomere organization compared to controls cultured using Matrigel-coated plates (Figure 2D). Whereas biological matrix (Figure 2C) facilitated sarcomere alignment in hiPSC-CMs, it did not affect the relative TNNI isoform profile during culture.

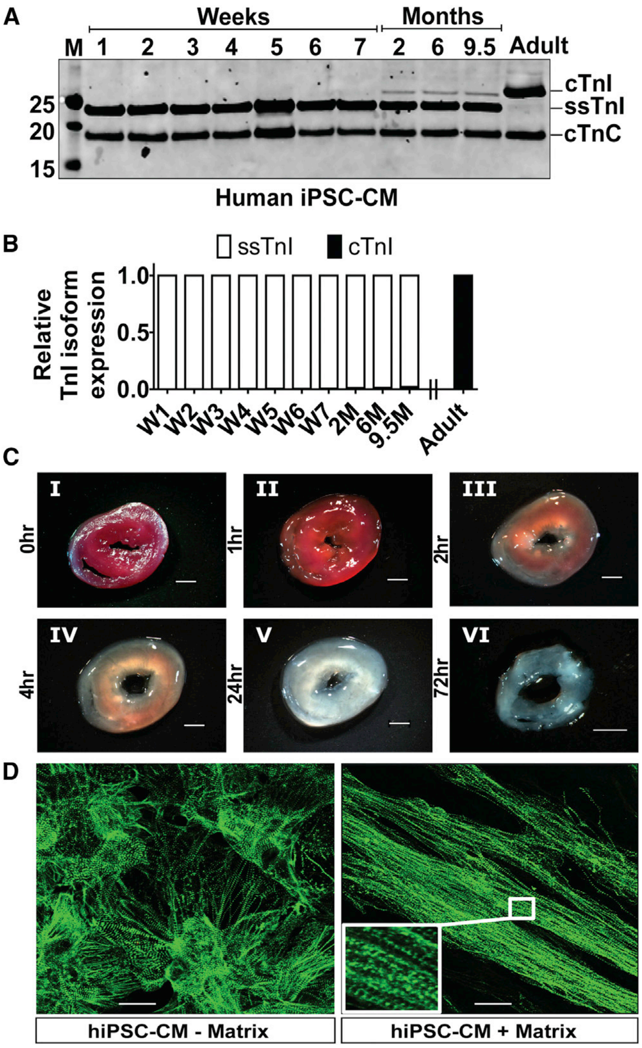

Figure 2. Temporal Expression Pattern of *TNNI* Protein Isoform Content and Sarcomeric Alignment of hiPSC-CMs by Biological Matrix

(A) Western blot data using pan TnI antibody showing dominant expression of ssTnI and little detectable cTnI expression even after culture of hiPSC-CMs spontaneously beating for 9.5 months. Adult human ventricular sample was used as positive control (far right lane).

(B) Summary bar graph of TnI isoform content in hiPSC-CMs during long-term culture in vitro (taken from western data in part A).

(C) Images of rat cardiac thin slice matrix constructs showing decellularization stages of transverse cardiac matrix isolated from whole heart. The whole heart was placed in stainless steel rat heart slicer and cut at 1 mm per thin slice before 1% SDS treatment (I) and 1 hr (II), 2 hr (III), 4 hr (IV), 24 hr (V), and 72 hr (VI) after 1% SDS treatment. Scale bar is 2 mm.

(D) Structural organization of hiPSC-CMs during culture with and without biological matrix. The images were taken with 40× objective; the calibration bar is 50 µm.

See also Figures S1, S3, and S4.

Temporal Expression Pattern of *TNNI* mRNA/Protein in Different hiPSC-CMs Lines

The stalled TnI isoform developmental transition we observed could have been specific to the DF19-9-11 hiPSC line or the differentiation protocol used. To address these possibilities, we performed companion studies using hiPSC-CMs obtained from multiple labs (see Experimental Procedures) and using different differentiation protocols. RNA and protein were extracted from five hiPSC-CMs lines at specific temporal points, including day 10, 2 weeks, 1 month, day 80, and 3 and 5 months. Whereas western blot analysis showed some cTnI induction in these additional lines, the stoichiometric ratio of cTnI:ssTnI remained very low and indicative of the immature state (Figure 3), similar to the DF19-9-11 hiPSC line (Figure 2).

Accelerated Acquisition of the Mature TnI Signature by T3 Supplementation in Rodent CMs, but Not hiPSC-CMs

The fetal gene program is regulated in response to developmental, hormonal, and hemodynamic stimuli (Parker et al., 1990). Thyroid hormone is critical in heart maturation during development (Chang et al., 1997; Trivieri et al., 2006; Mourouzis et al., 2011). Thus, we tested the direct effect of T3 on the *TNNI* isoform switch in hiPSC-CMs by supplementing media with 10 nM triiodothyronine (T3) for 2 weeks starting at 2 months postbeating of hiPSC-CMs (Figure 4). The 2 months postbeating time point was used for hiPSC-CMs because cTnI expression at this time point is comparable to cTnI expression profile at day 1 of rodent CMs (Figures 1D, 1F, and 1H). In parallel studies, hiPSC-CM media was supplemented with T3 at the onset of beating to compare to rodent CMs (Figure 4A). For comparison, day 1 Mesp1-mESC-CMs, M-NVCMs, and R-NVCMs (see Experimental Procedures) were supplemented with T3 for 2 weeks. Western blots showed the TnI isoform profile in hiPSC-CMs was not affected by T3 treatment (Figure 4), whereas Mesp1-mESC-CMs and rodent NVCMs responded significantly to T3 supplementation with Mesp1-mESC-CM expressing 95% and M-NVCM 98% cTnI post-T3 (Figures 4B–4D).

Acquisition of the Mature Signature by Stoichiometric Gene Replacement

Because hiPSC-CMs had stalled acquisition of the mature molecular signature, we sought to augment hiPSC-CM with exogenous adenoviral mediated gene transfer of human cTnI (Figure 5A). To test for acute expression of human cTnI on hiPSC-CMs, we generated adenovirus expressing cTnI and transduced hiPSC-CMs in serum-free primary culture conditions and assessed the expression of cTnI by western blotting. Post-cTnI gene transduction,

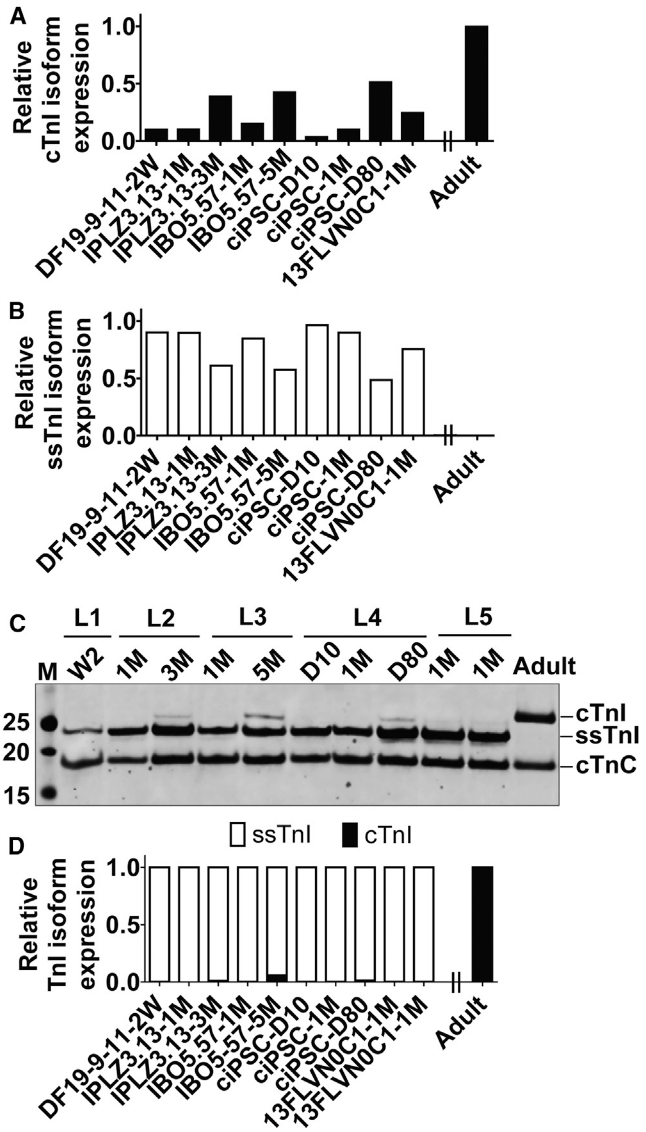

Figure 3. Temporal Expression Pattern of *TNNI* Isoform mRNA and Protein in Different hiPSC-CMs Lines

(A–C) The temporal developmental transition of ssTnI to cTnI was analyzed at the mRNA level to evaluate the transcriptional regulation in vitro in five hiPSC-CMs lines (A and B). RNA samples were prepared from five hiPSC-CMs lines obtained from different labs employing different differentiation conditions. The analyzed temporal points include day 10, 2 weeks, 1 month, day 80, and 3 and 5 months. A ventricle from an adult heart was used as a control. Quantitative RT-PCR data shows slight increase in the level of cTnI and concomitant reduction in ssTnI for hiPSC-CMs cultured for day 80 and 3 and 5 months in vitro (A and B). Data shown are expressed from average of (two) biological replicates. Western blot data using a pan TnI antibody showing the dominant expression of ssTnI and small yet detectable cTnI expression after culture of hiPSC-CMs for day 80 and 3 and 5 months in vitro (C).

(D) Summary of TnI isoform content in hiPSC-CMs obtained from different lines (taken from western data in part C). cTnC is used as cardiac-specific loading control and for normalization of samples for quantification purposes. Data shown are expressed from average of (two) biological replicates.

hiPSC-CMs had 80% expression of cTnI compared to the undetected cTnI content in nontransduced hiPSC-CMs (Figures 5B–5D). The level of ssTnI in cTnI transduced hiPSC-CMs was correspondingly reduced, consistent with stoichiometric sarcomere replacement. To assess if the observed effect is species dependent, we also transduced R-NVCM with a similar protocol used for hiPSC-CMs (Figure S5A). We observed 85% expression of cTnI 7 days posttransduction of R-NVCM compared to nontransduced controls (Figure S5B and S5C). The level of ssTnI was also significantly reduced compared with the nontransduced control (Figures S5B and S5D).

Acquisition of Chamber-Specific Markers in hiPSC-CMs

We used myosin light chain (MLC) 2a and 2v, atrial and ventricular chamber markers, respectively, to further track the identity and assess the *TNNI* isoform maturation gene switch in hiPSC-CMs. We employed IF to localize the expression of MLC2a and MLC2v at 20 and 60 days post-continuous spontaneous beating of hiPSC-CMs. At day 20, very few hiPSC-CMs were positive by IF for MLC2v as the vast majority of hiPSC-CM expressed MLC2a (Figures S4A–S4C). Interestingly, by day 60, MLC2v expression was robust while MLC2a expression was decreased (Figures S4D–S4F). Next, we used IF to evaluate whether MLC2v (ventricular marker) and cTnI (mature marker) were colocalized in 60 days post-spontaneous beating hiPSC-CM. Immunofluorescence and laser confocal scanning analysis demonstrated that hiPSC-CMs express primarily MLC2v with sparse cTnI detection (Figure S3). In day 20 rodent CMs, cTnI exclusively colocalized with MLC2v (Figure S3). For day 20 Mesp1-mESC-CMs, cTnI colocalized with MLC2v, suggesting mature ventricular cells with some cTnI-expressing myocytes that did not colocalize with MLC2v, suggesting these are atrial mature myocytes (Figure S3). These data also underscore the limitation of using IF as a mechanism to document *TNNI* isoform switching as compared to the quantification possible by western blotting (Figures 1, 2, 3, 4, and 5).

DISCUSSION

There is tremendous interest and many recent advances in the dynamic field of cardiac stem cell biology. Major breakthroughs in iPSC and reprogramming technologies further enable the potential of stem cell-based therapeutics in heart disease (Takahashi et al., 2007; Ieda et al., 2010). Before

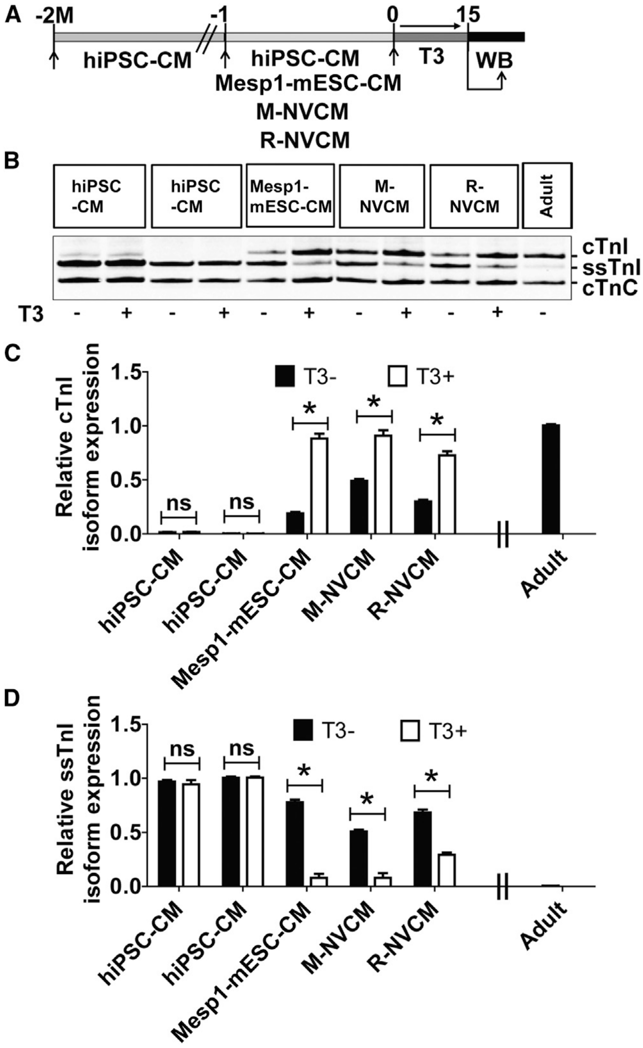

Figure 4. Accelerated Acquisition of the Mature *TNNI* Isoform Signature by T3 Supplementation in Rodent CMs, but Not in hiPSC-CMs

(A) Schematic of experimental time line for T3 supplementation in hiPSC-CM and rodent CMs. Two experimental time lines were used for hiPSC-CMs. The first was at 2 months postinitiation of beating, and the second time point was shortly after initiation of beating.

(B) Representative western blots TnI isoform profile detection after T3 supplementation (10 nM of T3 for 2 weeks in culture of 2 months beating hiPSC-CMs or early time point [1–2 days beating hiPSC-CMs] or 1 day rodent CMs).

(C and D) The effect of T3 treatment (T3+) or its absence (T3−) on acquisition of cTnI is quantified in (C) and suppression of ssTnI is quantified in (D). cTnC is used as cardiac-specific loading control and for normalization. Data analyzed by two-tailed t test and expressed as mean ± SEM, n = 3 independent experiments per group, $^*p < 0.05$.

potential can be fully realized, several key basic questions remain, including cell lineage assignment and maturation status of the myocytes. A general consensus in the field is that stem cell-derived myocytes are immature; however, there are no widely accepted markers to define and track the precise differentiation status of the emerging myocytes. Accordingly, the field would be advanced by establishing a molecular signature for tracking the differentiation status of stem cell-derived cardiac myocytes. In this light, we propose that the developmentally controlled and irreversible genetic switch in the sarcomeric *TNNI* genes provides an excellent marker to track the differentiation status of stem cell-derived cardiac myocytes. Owing to strict developmental control in all mammalian hearts, including humans, the programmed inactivation of the "fetal" *TNNI* gene product, ssTnI, together in exquisite temporal concert with stoichiometric replacement by the adult gene product, cTnI, represents a unique molecular marker of cardiac myocyte maturation status (Bhavsar et al., 1991).

Tracking the maturation status of stem cell-derived cardiac myocytes by ratiometric analysis of the cTnI:ssTnI protein profile provides a tool to permit direct comparisons of myocyte developmental status from different cell lines and with using different culture methods and differentiation protocol across laboratories. Tracking the cTnI/ssTnI isoform expression profile will be useful in cataloging the large array of other pertinent markers of myocyte development including molecular, structural, electrical, morphological, and functional features of maturing stem cell-derived cardiac myocytes. Ultimately, to be defined as an adult myocyte would require acquisition of the well-known structural and functional profile of the mature myocyte, and we propose the adult designation would require the exclusive expression of the cTnI isoform (Bhavsar et al., 1991; Siedner et al., 2003).

Key features of the TnI protein isoform switch as a quantitative standard for cardiac stem cell-derived myocyte maturation are (1) its stoichiometric conservation, (2) its normal developmental irreversibility, and (3) its physiological significance (Siedner et al., 2003; Davis et al., 2008). Conservation of a strict one-to-one stoichiometric expression pattern provides a unique internal standard on which the cTnI:ssTnI isoform ratio informs a direct quantitative marker of development. This differs from tracking, for example, the relative abundance of a single cardiac marker in developing cells, such as cardiac actin, α-actinin, or myosin light chains, because a reference internal "fetal" standard is lacking thus limiting their use. Further, often used cardiac development markers such as cell morphology and striation appearance are inherently subjective in terms of precise tracking and cataloging maturation progression. Irreversibility in the ssTnI to cTnI transition is of value as a maturation marker. At the level of the cardiac sarcomere, as cTnI (*TNNI3* gene product) progressively replaces ssTnI (*TNNI1)* during maturation, there is no reactivation of the fetal *TNNI1* gene program, even in disease states

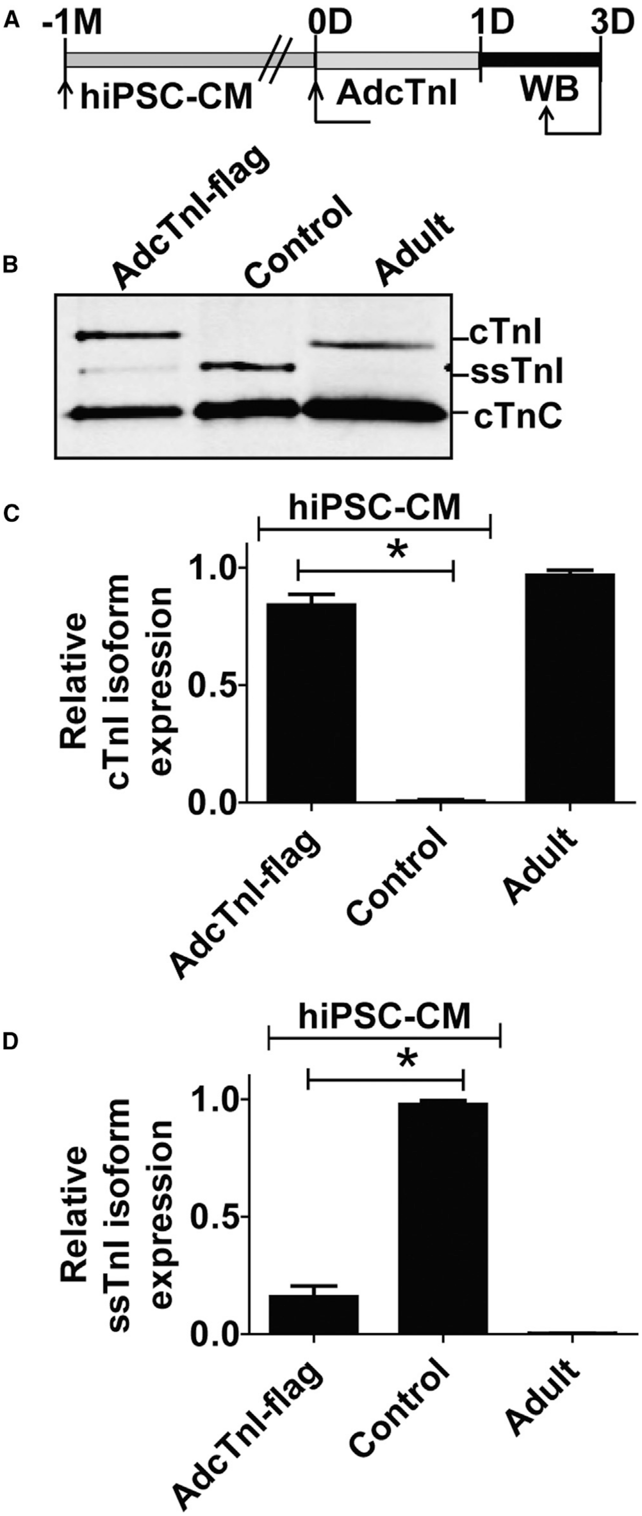

Figure 5. Acquisition of the Mature *TNNI* Isoform Signature by Gene Transfer in hiPSC-CMs

(A) Experimental time line for AdcTnI (flag tagged) gene transfer.
(B–D) Western blot probed with pan TnI isoform antibody (B) and summary bar graphs of relative cTnI expression (C) and ssTnI expression (D) in hiPSC-CMs. cTnC is used as cardiac-specific loading control and for normalization of samples for quantification purposes. See also Figure S5. Values are analyzed by one-way ANOVA with Bonferroni post hoc test and expressed as mean ± SEM, n = 3 independent experiments, $*p < 0.05$.

including stress, hypertrophy (physiological or maladaptive), ischemia, or in heart failure (Bhavsar et al., 1991; Solaro, 1999). In all mammalian hearts, including human, it is the complete stoichiometric replacement of ssTnI by cTnI that is as an immutable profile of acquiring the mature adult state. Accordingly, to be termed adult, a cardiac myocyte requires a profile of 100% cTnI and 0% (i.e., undetectable) ssTnI. It would be insufficient, for example, to simply detect cTnI expression (as a single cardiac marker by IF or fluorescence-activated cell sorting [FACS]) to track differentiation status, without also documenting in unison the complete stoichiometric loss of ssTnI expression. We propose the following cTnI:ssTnI protein isoform ratio, quantified by western blot analysis (with a pan TnI isoform antibody as shown here), be implemented for the developmental stage classification of populations of stem cell-derived cardiac myocytes: immature = 0:100 to 15:85; neonatal-like = >15: <85 to 90: 10; mature profile = 100:0 (Figure 6). Other techniques such as IF or FACS would not be adequate for quantifying this TnI isoform profile because of limitations in precise ratiometric analysis of the stoichiometrically conserved cTnI:ssTnI isoform ratio.

Numerous cardiac markers, including molecular, structural, and physiological, have been used in attempt to track and classify cardiac cells derived from stem cell progenitors or from direct reprogramming (Ieda et al., 2010; Qian et al., 2012). The sarcomere markers cTnT and myosin are in frequent use, along with the detection of myofilament striations, myocyte morphology, Ca^{2+} handling, rhythmic beating, action potential shape, and others for cardiac lineage assignment and development assessment (Radisic et al., 2004; Lundy et al., 2013). Limitations of these markers include questions of lineage specificity (e.g., cTnT is also expressed in smooth muscle) and developmental progression. For example, markers such as TnT and MyHC show isoform developmental regulation with the complicating feature of reversion to the "fetal" program in disease (Parker et al., 1990; Kinugawa et al., 2001). The failing adult cardiac myocyte reactivates the fetal program at many levels, including structural, hormonal, and metabolic gene profile by downregulating adult gene transcripts (Parker et al., 1990; Kinugawa et al., 2001). For instance, transcript levels of *ANF*, *BNP*, skeletal actin, *βMHC*, and metabolic genes such as *GLUT1* are higher in the human fetal and failing hearts than in the normal adult heart (Pasternac and Cantin, 1990; Tanaka et al., 1995; Shioi et al., 2000). In concert, transcript levels of adult genes such as

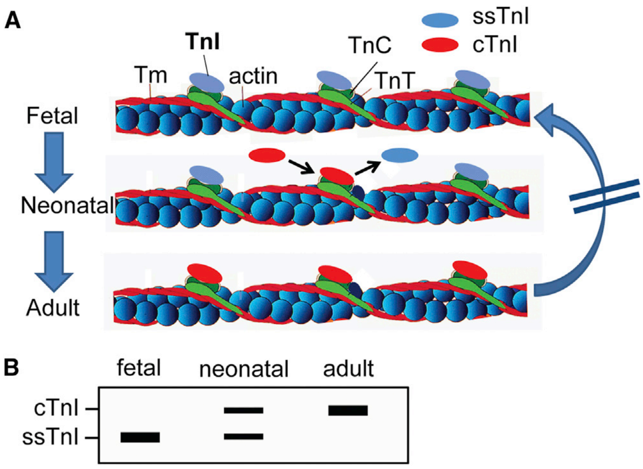

Figure 6. *TNNI* Protein Isoform Switch as a Quantitative Maturation Marker for Stem Cell-Derived Cardiac Myocytes
(A) Schematic of the cardiac sarcomere thin filament illustrating the stoichiometrically conserved transition from the fetal TnI (ssTnI) to the adult TnI isoform (cTnI) profile. Owing to strict protein content control in the sarcomere, the ssTnI isoform must be removed before the cTnI isoform can occupy the same position in perfect 1:1 synchrony during development. At any time in development, the transition from ssTnI to cTnI is definitive in that it cannot revert (blocked arrow on right).
(B) Hypothetical western blot based on published work from Sasse et al. (1993) depicting the TnI isoform profile in cardiac development.

αMHC and *SERCA-2a*, ion channels, and metabolic genes such as *GLUT4* are reduced in failing human heart (Pasternac and Cantin, 1990; Tanaka et al., 1995; Lowes et al., 1997; Shioi et al., 2000). Previous studies have used one or more of these genes to evaluate maturation of in vitro derived cardiac myocytes (Radisic et al., 2004; Chan et al., 2013b; Lundy et al., 2013). Therefore, it cannot be certain by testing, for example, MyHC isoforms or Ca^{2+} handling function that the myocytes in question are at a fetal stage of development or rather are reverting in profile owing to latent activation of the fetal program. In other words, markers in frequent use today in cardiac stem cell biology are highly plastic in nature, making difficult any specific assignment of myocyte maturation. As mentioned, because cardiac TnT is also expressed in smooth muscle cells, the use of cTnT to quantify CMs must be interpreted with caution. Therefore, it is important to incorporate at least a second cardiac-specific marker for cardiac lineage assignment. Equally important, the use of MLC2a and MLC2v to define maturation is not straightforward. This is because although at early developmental time points most hiPSC-CMs express MLC2a and not MLC2v, at later time points, they express robust MLC2v, with MLC2a still significantly expressed. To be classified as a mature ventricular myocyte, stem cell-derived CMs require 100% cTnI (and 0% ssTnI) that is colocalized with MLC2v. MLC2v alone cannot serve as a mature marker in CMs, as we show MLC2V-positive myocytes express primarily ssTnI (the immature marker) (Figure S4).

In our long-term cultures of human iPSC CMs, the cTnI:ssTnI ratio stalled at ~2:98, making it fetal-like with regard to the TnI isoform profile. This stalled marker of development was observed using different differentiation protocols, in multiple hiPSC lines (five hiPSC-CMs line obtained from different labs) and over long-term culture (9+ months). In an attempt to modify this ratio via extrinsic signaling mechanisms, we employed varied media modifications including thyroid hormone supplementation. In rodent myocytes in culture, T3-supplemented media accelerated the transition from ssTnI to cTnI; however, in human iPSC-CMs, no change in the TnI isoform ratio was obtained. The basis for the lack of effect of T3 on hiPSC-CMs to influence changes in TnI isoforms is not clear. Recent studies have shown T3 to have an effect on αMHC and SERCA2a expression profiles in hiPSC-CMs, indicating that key components of the T3 signaling cascade are present (Ivashchenko et al., 2013; Yang et al., 2014). The only successful method used here to more closely approach the mature adult TnI signature was via direct gene transfer of *TNNI3*, which was highly efficient in stoichiometric replacement of ssTnI with cTnI, similar in practice to our previous work in adult cardiac myocytes (Westfall et al., 1997; Day et al., 2006).

Along with implementing TnI isoform ratio as a maturation marker, it is well known that TnI isoforms have significant physiological implications for cardiac myocyte performance (Siedner et al., 2003; Davis et al., 2008). There is considerable debate regarding identifying the specific properties of stem cell-derived cardiac myocytes most suitable in terms of effecting physiologically meaningful regeneration/repair in disease myocardium in vivo. In terms of the profile of TnI isoform expression, there are positive and negatives attributed to the fetal and adult forms of the *TNNI* gene. The *TNNI1* gene product, ssTnI, has unique molecular switch properties that would be expected to be an advantage in terms of survival in the harsh environment where stem cell-derived myocytes are placed upon transfer to ischemic and diseased hearts. Here, the ssTnI protein confers robust Ca^{2+} activated contraction in an environment where blood supply is inadequate and in the biochemical milieu of acidosis (Westfall et al., 1997; Day et al., 2006), as would be expected during cellular transplantation. A negative aspect of ssTnI function relates to its detrimental effects on relaxation performance owing to comparatively slow inactivation of the thin filament in diastole (Davis et al., 2008). In comparison, the adult *TNNI3* gene product, cTnI, by virtue of its unique 32-amino-acid N-terminal extension containing target

PKA-responsive serines, confers enhanced relaxation performance to the myofilaments that is physiologically important to overall normal heart performance (Davis et al., 2008). However, a negative attribute of cTnI is its marked downregulation in function in ischemic/hypoxic conditions that would be expected in the milieu of the transplanted myocytes (Westfall et al., 1997; Day et al., 2006). In this light, the ideal TnI molecule would retain the best properties of both cTnI and ssTnI isoforms. We have used structure-function and evolutionary biology concepts to guide construction of a unique *TNNI* gene product that incorporates an acidosis resistant histidine button while retaining fast inactivation properties required to cardiac lusitropy during adrenergic stimulation (Day et al., 2006). We would propose this engineered molecular switch protein to be ideal in genetic design of stem cell-derived cardiac myocytes for repair in vivo.

While the TnI isoform ratio is proposed here to be necessary for tracking maturation of stem cell-derived cardiac myocyte, it is not sufficient, on its own, to establish the mature adult cardiac myocyte state. Many other well-defined physiological and structural markers must also assemble in concert to fully define a myocyte as adult, including highly refined Ca^{2+} handling, exquisite cell morphology and subcellular structure, and unique physiological forces and motion. Ultimately, these features, which are critical but limited by their restricted use in isolation (owing to issues of fetal-like reversion in stress and disease states), make the TnI switch profile valuable as an immutable barometer to gauge methods seeking to advance stem cell-derived myocytes toward the mature adult state. Mindful of the complexities listed above, the cTnI:ssTnI protein isoform ratio will enable a differentiation status standard on which cardiac myocytes can be directly compared between research groups as we have tested in this study. This could be useful in cardiac stem cell myocyte applications in the lab and the clinic.

EXPERIMENTAL PROCEDURES

Isolation and Culture of Rodent CMs

Animals used in these experiments were handled in accordance with guide lines set by the Institutional Animal Care and Use Committees (IACUC) of University of Minnesota protocol approval. For in vitro culture experiments, hearts were obtained from P0-P1 Sprague-Dawley rat pups. Rat neonatal ventricular cardiac myocytes (R-NVCM) were isolated using sequential enzyme digestion as described in the isolation kit (Worthington). R-NVCMs were plated either onto poly-D-lysine-coated coverslips or poly-D-lysine-coated + matrix coverslips for 1–2 hr. Medium was changed every 2–3 days. A similar protocol was used for the isolation and in vitro culture of mouse neonatal ventricular cardiac myocytes (M-NVCM).

Isolation of Hearts from Rodent Pups

For in vivo developmental study, hearts from pups were isolated at different developmental time points: P1, P3, P5, P7, P10, P15, and P20. Protein or RNA was isolated from each hearts at different time points indicated and used for western blotting, mRNA expression, and immunohistochemistry. Animals used in these experiments were handled in accordance with guideline set by the IACUC of University of Minnesota protocol approval.

Maintenance of hiPSCs and Differentiation into Cardiac Myocytes

The primary hiPSC line used in this study was DF 19-9-11 (derived from neonatal skin fibroblast) and was maintained on feeder-free Matrigel (BD Biosciences) in mTeSR1 or TeSR-E8 medium (STEM CELL Technologies) (Figure S1A). For cardiac differentiation via small molecules, as adapted from Lian et al. (2012), hiPSCs were maintained on Matrigel plates for 4 days or until they reached confluence. Cells were treated with 10 μM CHIR99021 (GSK-3 inhibitor, Selleck Bio) along with Matrigel in RPMI/B27 without insulin (Invitrogen) for 24 hr (day 0 to day 1). The next day, medium was changed to RPMI/B27 without insulin plus inhibitor of Wnt protein 4 (IWP4) at 5 μM (Stemgent) and removed during the medium change on day 2. Cells were maintained in the RPMI/B27 plus insulin starting from day 7, with media changes every 1–2 days (Figures S1A and S1B). The use of RPMI and B27+ insulin cocktail culture condition has the advantage of preventing fibroblast overgrowth compared to use of FBS. Regular media changes were performed to limit pH alterations.

HiPSC-CMs Using Different Lines and Differentiation Protocols

Line 1 hiPSCs (DF19-9-11 hiPSCs) (derived from [neonatal] skin fibroblasts) were maintained in culture for 4–5 days on Matrigel (BD Biosciences) and mTeSR medium (STEMCELL Technologies). Differentiation was initiated as described previously (Zhang et al., 2012) by adding cold RPMI/B27 minus insulin containing Matrigel (0.5 mg/plate) and 100 ng/ml Activin A (R&D Systems) at day 0. At day 1, medium was exchanged with RPMI/B27 minus insulin supplement containing 5 ng/ml BMP4 (R&D Systems) + 5 ng/ml bFGF (Invitrogen) until day 5. Starting at day 5, differentiating hiPSCs were fed with medium containing RPMI/B27 complete supplement (+ insulin) at interval of 2–3 days. Protein and RNA samples were collected at day 15 postinitiation of beating.

Lines 2 and 3 hiPSCs (IPLZ3.13 and IBO5.57 hiPSC) (derived from skin fibroblasts) were maintained in culture for 4–5 days on Matrigel (BD Biosciences) and mTeSR medium (STEMCELL Technologies). Differentiation was initiated as described previously (Zhang et al., 2012) by adding cold RPMI/B27 minus insulin (Invitrogen) containing Matrigel (0.5 mg/plate) and 100 ng/ml Activin A (R&D Systems) and 10 μM CHIR-99021 (Selleck Bio) at day 0. At day 1, medium was exchanged with RPMI/B27 minus insulin supplement containing 5 ng/ml BMP4 + 5 ng/ml bFGF (R&D Systems). At day 3, IWP4 (Stemgent) was supplemented at final concentration of 5 μM. Starting at day 5, differentiating hiPSCs were fed with medium containing RPMI/B27 complete supplement (+ insulin) at interval of 2–3 days. Protein and RNA samples were collected at day 30, 3 months, and 5 months.

Line 4 hiPSCs (hciPSCs) (derived from cardiac fibroblast) were maintained in culture for 4–5 days on Matrigel and mTeSR medium. Differentiation was initiated into CMs via the Matrigel sandwich method, as described previously (Zhang et al., 2012). Briefly, 1×10^6 single hiPSCs were expanded on a Matrigel-coated dish for 3–5 days, and then differentiation was induced on day 0 by culturing the cells with 100 ng/ml Activin A (R&D Systems) in RPMI basal medium plus B27 without insulin. Twenty-four hours later (i.e., on day 1 of differentiation), cells were treated with 10 ng/ml BMP4 and 7.5 ng/ml bFGF (R&D Systems) in RPMI basal medium plus B27 without insulin. Four days later (i.e., on day 5 of differentiation), cells were cultured in RPMI basal medium with normal B27, and beating cells usually appeared approximately on day 8 after differentiation was initiated. Protein and RNA samples were collected at day 10, day 30, and day 80.

Line 5 hiPSCs (13FLVN0C1 hiPSCs) (derived from skin fibroblasts) were maintained in culture for 5 days on Matrigel (BD Biosciences) and TeSR-E8 medium. Differentiation was initiated as described previously (Lian et al., 2012) by adding 12 μM CHIR-99021 with RPMI/B27 minus insulin supplement at day 0. At day 3, IWP2 was added at final concentration of 5 μM. At day 7, differentiating hiPSCs were fed with medium containing RPMI/B27 complete supplement (+ insulin) at interval of 2–3 days. Protein and RNA samples were collected at 1 month after initiation of beating.

Generation of Inducible Mesp1-mESC and Their Differentiation into Cardiac Myocytes

mESC-derived cardiac myocytes were obtained using a doxycycline-inducible Mesp1 mESC line (Chan et al., 2013a). For cardiac differentiation, embryoid bodies (EBs) were aggregated by seeding 0.5 million single cells per 10 ml in a petri dish with orbital shaking (70 rpm) (day 0) (Figure S1C). Media changes were on days 3 and 4. Doxycycline was added on day 3 at a final concentration of 500 ng/ml and removed on day 4. IWR1 was added on day 3 at a final concentration of 10 μM. EB cells were harvested on day 6. Beating EBs were dissociated using trypsin to obtain single cardiac myocytes. Trypsinized individualized cardiac myocytes were seeded on coverslips coated with either poly-D-lysine or poly-D-lysine plus cardiac matrix at cell density of 150–200,000 cells per coverslip.

T3 Supplementation in Culture of hiPSC-CMs and Rodent CMs

The possible direct effect of T3 (Sigma) on the *TNNI* isoform switching was tested by supplementing T3 (10 nM) for 2 weeks in cultures of 2 months postbeating hiPSC-CMs and at early time points shortly after the onset of beating (day 1 postbeat hiPSC-CMs). Similarly, day 1 Mesp1-mESC-CM, M-NVCM, and R-NVCM were supplemented with T3 for 2 weeks. The expression of cTnI was assessed by western blotting on protein samples collected 15 days after treatment.

Transduction of hiPSC-CMs or Rodent CMs with Adenoviral Vectors

HiPSC-CMs or rodent-CMs were transduced with recombinant adenovirus vectors harboring cTnI(C-terminal flag tagged) in serum-free medium for 2–3 hr at multiplicity of infection of 100–200. The transduced hiPSC-CMs were maintained in culture with RPMI supplemented with B27 plus insulin.

Western Blotting and Indirect Immunohistochemistry

Cardiac myocytes were lysed in RIPA buffer containing protease and phosphatase inhibitor mixture. Proteins were separated by 10% (wt/vol) *Tris*Glycine SDS/PAGE (Invitrogen) under denaturing conditions and transferred to a nitrocellulose membrane. After blocking with 5% milk Tris-buffered saline Tween-20 (TBST) for 1 hr, the membrane was incubated with primary antibody overnight at 4°C. The membrane was washed, incubated with an anti-mouse/rabbit fluorescent dye-conjugated secondary antibody (1:5,000, Invitrogen) at room temperature for 1 hr and scanned by Odyssey (Bio-Rad). Primary antibodies used included mouse raised pan-TnI specific (MAB1691), cTnC, MLC2a and rabbit raised TnT, MLC2v. Blocked samples were probed with pan TnI specific (MAB1691), cTnC (1A2), cTnI (4C2), and MLC2a primary antibody each raised in mouse (1:1,000 dilution) or TnT, ssTnI, and MLC2v primary antibody each raised in rabbit (1:1,000 dilution). cTnC raised in mouse (clone 1A2 Abcam) was used to evaluate protein loading. The binding of primary antibodies was detected by goat anti-rabbit (IRDye 800 conjugates) (Rockland Immunochemicals) or goat anti-mouse (Alexa Fluor 680 conjugates) (Invitrogen) secondary antibodies (1:5,000) and scanned with LI-COR Odyssey infrared imaging system (LI-COR Biosciences).

For indirect immunohistochemistry, hiPSC-CMs were fixed in 4% paraformaldehyde for 5–10 min at room temperature and permeabilized with 0.25% Triton X-100 in PBS. Permeabilized cells were blocked with 20% goat serum. Blocked myocytes were probed with primary antibodies specific for anti-myosin light chain MLC2a (1:1,000, Synaptic Systems), cTnI (4C2) and sarcomeric actinin (EA-53) raised in mouse (1:1,000, Sigma), and MLC2v, ssTnI, and TnT raised in rabbit (1:1,000 dilution). The binding of primary antibodies was visualized with goat anti-mouse immunoglobulin G conjugated to Alexa 488 (1:1,000, Sigma) and goat anti-rabbit conjugated to Alexa Fluor 594 secondary antibodies (1:1,000). Representative cells were photographed using a Zeiss confocal microscope.

Statistical Analysis

Data are expressed as mean ± SEM with Student's t test to determine statistical significance. For comparison among three or more treatment groups, one-way ANOVA was used. For comparison among treatment groups with two independent variables, two-way ANOVA was used. $p < 0.05$ was considered statistically significant.

SUPPLEMENTAL INFORMATION

Supplemental Information includes Supplemental Experimental Procedures and five figures and can be found with this article online at http://dx.doi.org/10.1016/j.stemcr.2014.07.012.

ACKNOWLEDGMENTS

We thank Dr. Joseph C. Wu (Stanford University) for providing some additional hiPSC-CMs for our study and Mr. Chris Chapman for his assistance. This work is supported by NIH PCBC U01

HL1000407 and HL099773 (D.J.G., T.J.K., J.Z., M.K., J.M.M., and J.C.W.), NIH R01 (J.M.M.), and R24 HL117756 (J.C.W.). We acknowledge support from the Lillihei Heart Institute to this study. T.J.K. is a founding shareholder and consultant for Cellular Dynamics International.

Received: February 14, 2014
Revised: July 22, 2014
Accepted: July 24, 2014
Published: September 4, 2014

REFERENCES

Averyhart-Fullard, V., Fraker, L.D., Murphy, A.M., and Solaro, R.J. (1994). Differential regulation of slow-skeletal and cardiac troponin I mRNA during development and by thyroid hormone in rat heart. J. Mol. Cell. Cardiol. *26*, 609–616.

Bhavsar, P.K., Dhoot, G.K., Cumming, D.V., Butler-Browne, G.S., Yacoub, M.H., and Barton, P.J. (1991). Developmental expression of troponin I isoforms in fetal human heart. FEBS Lett. *292*, 5–8.

Chan, S.S., Shi, X., Toyama, A., Arpke, R.W., Dandapat, A., Iacovino, M., Kang, J., Le, G., Hagen, H.R., Garry, D.J., and Kyba, M. (2013a). Mesp1 patterns mesoderm into cardiac, hematopoietic, or skeletal myogenic progenitors in a context-dependent manner. Cell Stem Cell *12*, 587–601.

Chan, Y.C., Ting, S., Lee, Y.K., Ng, K.M., Zhang, J., Chen, Z., Siu, C.W., Oh, S.K., and Tse, H.F. (2013b). Electrical stimulation promotes maturation of cardiomyocytes derived from human embryonic stem cells. J. Cardiovasc. Transl. Res. *6*, 989–999.

Chang, K.C., Figueredo, V.M., Schreur, J.H., Kariya, K., Weiner, M.W., Simpson, P.C., and Camacho, S.A. (1997). Thyroid hormone improves function and Ca2+ handling in pressure overload hypertrophy. Association with increased sarcoplasmic reticulum Ca2+-ATPase and alpha-myosin heavy chain in rat hearts. J. Clin. Invest. *100*, 1742–1749.

Davis, J., Westfall, M.V., Townsend, D., Blankinship, M., Herron, T.J., Guerrero-Serna, G., Wang, W., Devaney, E., and Metzger, J.M. (2008). Designing heart performance by gene transfer. Physiol. Rev. *88*, 1567–1651.

Day, S.M., Westfall, M.V., Fomicheva, E.V., Hoyer, K., Yasuda, S., La Cross, N.C., D'Alecy, L.G., Ingwall, J.S., and Metzger, J.M. (2006). Histidine button engineered into cardiac troponin I protects the ischemic and failing heart. Nat. Med. *12*, 181–189.

Dhoot, G.K., Gell, P.G., and Perry, S.V. (1978). The localization of the different forms of troponin I in skeletal and cardiac muscle cells. Exp. Cell Res. *117*, 357–370.

Ebashi, S. (1983). Regulation of muscle contraction. Cell Muscle Motil. *3*, 79–87.

Ebashi, S. (1988). Historical overview. Calcium ion and contractile proteins. Ann. N Y Acad. Sci. *522*, 51–59.

Hansson, E.M., and Chien, K.R. (2012). Reprogramming a broken heart. Cell Stem Cell *11*, 3–4.

Hazeltine, L.B., Badur, M.G., Lian, X., Das, A., Han, W., and Palecek, S.P. (2013). Temporal impact of substrate mechanics on differentiation of human embryonic stem cells to cardiomyocytes. Acta Biomater. *10*, 604–612.

Huang, X., Lee, K.J., Riedel, B., Zhang, C., Lemanski, L.F., and Walker, J.W. (2000). Thyroid hormone regulates slow skeletal troponin I gene inactivation in cardiac troponin I null mouse hearts. J. Mol. Cell. Cardiol. *32*, 2221–2228.

Hunkeler, N.M., Kullman, J., and Murphy, A.M. (1991). Troponin I isoform expression in human heart. Circ. Res. *69*, 1409–1414.

Ieda, M., Fu, J.D., Delgado-Olguin, P., Vedantham, V., Hayashi, Y., Bruneau, B.G., and Srivastava, D. (2010). Direct reprogramming of fibroblasts into functional cardiomyocytes by defined factors. Cell *142*, 375–386.

Ivashchenko, C.Y., Pipes, G.C., Lozinskaya, I.M., Lin, Z., Xiaoping, X., Needle, S., Grygielko, E.T., Hu, E., Toomey, J.R., Lepore, J.J., and Willette, R.N. (2013). Human-induced pluripotent stem cell-derived cardiomyocytes exhibit temporal changes in phenotype. Am. J. Physiol. Heart Circ. Physiol. *305*, H913–H922.

Kawamura, M., Miyagawa, S., Fukushima, S., Saito, A., Miki, K., Ito, E., Sougawa, N., Kawamura, T., Daimon, T., Shimizu, T., et al. (2013). Enhanced survival of transplanted human induced pluripotent stem cell-derived cardiomyocytes by the combination of cell sheets with the pedicled omental flap technique in a porcine heart. Circulation *128* (11 Suppl 1), S87–S94.

Kinugawa, K., Minobe, W.A., Wood, W.M., Ridgway, E.C., Baxter, J.D., Ribeiro, R.C., Tawadrous, M.F., Lowes, B.A., Long, C.S., and Bristow, M.R. (2001). Signaling pathways responsible for fetal gene induction in the failing human heart: evidence for altered thyroid hormone receptor gene expression. Circulation *103*, 1089–1094.

Lehman, W., Rosol, M., Tobacman, L.S., and Craig, R. (2001). Troponin organization on relaxed and activated thin filaments revealed by electron microscopy and three-dimensional reconstruction. J. Mol. Biol. *307*, 739–744.

Lian, X., Hsiao, C., Wilson, G., Zhu, K., Hazeltine, L.B., Azarin, S.M., Raval, K.K., Zhang, J., Kamp, T.J., and Palecek, S.P. (2012). Robust cardiomyocyte differentiation from human pluripotent stem cells via temporal modulation of canonical Wnt signaling. Proc. Natl. Acad. Sci. USA *109*, E1848–E1857.

Lowes, B.D., Minobe, W., Abraham, W.T., Rizeq, M.N., Bohlmeyer, T.J., Quaife, R.A., Roden, R.L., Dutcher, D.L., Robertson, A.D., Voelkel, N.F., et al. (1997). Changes in gene expression in the intact human heart. Downregulation of alpha-myosin heavy chain in hypertrophied, failing ventricular myocardium. J. Clin. Invest. *100*, 2315–2324.

Lu, T.Y., Lin, B., Kim, J., Sullivan, M., Tobita, K., Salama, G., and Yang, L. (2013). Repopulation of decellularized mouse heart with human induced pluripotent stem cell-derived cardiovascular progenitor cells. Nat. Commun. *4*, 2307.

Lundy, S.D., Zhu, W.Z., Regnier, M., and Laflamme, M.A. (2013). Structural and functional maturation of cardiomyocytes derived from human pluripotent stem cells. Stem Cells Dev. *22*, 1991–2002.

MacGeoch, C., Barton, P.J., Vallins, W.J., Bhavsar, P., and Spurr, N.K. (1991). The human cardiac troponin I locus: assignment to chromosome 19p13.2-19q13.2. Hum. Genet. *88*, 101–104.

Mahmoud, A.I., Kocabas, F., Muralidhar, S.A., Kimura, W., Koura, A.S., Thet, S., Porrello, E.R., and Sadek, H.A. (2013). Meis1 regulates postnatal cardiomyocyte cell cycle arrest. Nature *497*, 249–253.

Metzger, J.M., and Westfall, M.V. (2004). Covalent and noncovalent modification of thin filament action: the essential role of troponin in cardiac muscle regulation. Circ. Res. *94*, 146–158.

Mourouzis, I., Forini, F., Pantos, C., and Iervasi, G. (2011). Thyroid hormone and cardiac disease: from basic concepts to clinical application. J. Thyroid Res. *2011*, 958626.

Nakao, K., Minobe, W., Roden, R., Bristow, M.R., and Leinwand, L.A. (1997). Myosin heavy chain gene expression in human heart failure. J. Clin. Invest. *100*, 2362–2370.

Ohtsuki, I., Maruyama, K., and Ebashi, S. (1986). Regulatory and cytoskeletal proteins of vertebrate skeletal muscle. Adv. Protein Chem. *38*, 1–67.

Palpant, N.J., and Murry, C.E. (2012). Regenerative medicine: Reprogramming the injured heart. Nature *485*, 585–586.

Park, H., Larson, B.L., Kolewe, M.E., Vunjak-Novakovic, G., and Freed, L.E. (2014). Biomimetic scaffold combined with electrical stimulation and growth factor promotes tissue engineered cardiac development. Exp. Cell Res. *321*, 297–306.

Parker, T.G., Packer, S.E., and Schneider, M.D. (1990). Peptide growth factors can provoke “fetal” contractile protein gene expression in rat cardiac myocytes. J. Clin. Invest. *85*, 507–514.

Parmacek, M.S., and Solaro, R.J. (2004). Biology of the troponin complex in cardiac myocytes. Prog. Cardiovasc. Dis. *47*, 159–176.

Pasternac, A., and Cantin, M. (1990). Atrial natriuretic factor: a ventricular hormone? J. Am. Coll. Cardiol. *15*, 1446–1448.

Porrello, E.R., Mahmoud, A.I., Simpson, E., Hill, J.A., Richardson, J.A., Olson, E.N., and Sadek, H.A. (2011). Transient regenerative potential of the neonatal mouse heart. Science *331*, 1078–1080.

Porrello, E.R., Mahmoud, A.I., Simpson, E., Johnson, B.A., Grinsfelder, D., Canseco, D., Mammen, P.P., Rothermel, B.A., Olson, E.N., and Sadek, H.A. (2013). Regulation of neonatal and adult mammalian heart regeneration by the miR-15 family. Proc. Natl. Acad. Sci. USA *110*, 187–192.

Qian, L., Huang, Y., Spencer, C.I., Foley, A., Vedantham, V., Liu, L., Conway, S.J., Fu, J.D., and Srivastava, D. (2012). In vivo reprogramming of murine cardiac fibroblasts into induced cardiomyocytes. Nature *485*, 593–598.

Radisic, M., Park, H., Shing, H., Consi, T., Schoen, F.J., Langer, R., Freed, L.E., and Vunjak-Novakovic, G. (2004). Functional assembly of engineered myocardium by electrical stimulation of cardiac myocytes cultured on scaffolds. Proc. Natl. Acad. Sci. USA *101*, 18129–18134.

Razeghi, P., Young, M.E., Alcorn, J.L., Moravec, C.S., Frazier, O.H., and Taegtmeyer, H. (2001). Metabolic gene expression in fetal and failing human heart. Circulation *104*, 2923–2931.

Rebuzzini, P., Fassina, L., Mulas, F., Bellazzi, R., Redi, C.A., Di Liberto, R., Magenes, G., Adjaye, J., Zuccotti, M., and Garagna, S. (2013). Mouse embryonic stem cells irradiated with γ-rays differentiate into cardiomyocytes but with altered contractile properties. Mutat. Res. *756*, 37–45.

Reiser, P.J., Portman, M.A., Ning, X.H., and Schomisch Moravec, C. (2001). Human cardiac myosin heavy chain isoforms in fetal and failing adult atria and ventricles. Am. J. Physiol. Heart Circ. Physiol. *280*, H1814–H1820.

Sasse, S., Brand, N.J., Kyprianou, P., Dhoot, G.K., Wade, R., Arai, M., Periasamy, M., Yacoub, M.H., and Barton, P.J. (1993). Troponin I gene expression during human cardiac development and in end-stage heart failure. Circ. Res. *72*, 932–938.

Shiba, Y., Fernandes, S., Zhu, W.Z., Filice, D., Muskheli, V., Kim, J., Palpant, N.J., Gantz, J., Moyes, K.W., Reinecke, H., et al. (2012). Human ES-cell-derived cardiomyocytes electrically couple and suppress arrhythmias in injured hearts. Nature *489*, 322–325.

Shioi, T., Kang, P.M., Douglas, P.S., Hampe, J., Yballe, C.M., Lawitts, J., Cantley, L.C., and Izumo, S. (2000). The conserved phosphoinositide 3-kinase pathway determines heart size in mice. EMBO J. *19*, 2537–2548.

Siedner, S., Krüger, M., Schroeter, M., Metzler, D., Roell, W., Fleischmann, B.K., Hescheler, J., Pfitzer, G., and Stehle, R. (2003). Developmental changes in contractility and sarcomeric proteins from the early embryonic to the adult stage in the mouse heart. J. Physiol. *548*, 493–505.

Solaro, R.J. (1999). Troponin I, stunning, hypertrophy, and failure of the heart. Circ. Res. *84*, 122–124.

Takahashi, K., Tanabe, K., Ohnuki, M., Narita, M., Ichisaka, T., Tomoda, K., and Yamanaka, S. (2007). Induction of pluripotent stem cells from adult human fibroblasts by defined factors. Cell *131*, 861–872.

Tanaka, M., Hiroe, M., Ito, H., Nishikawa, T., Adachi, S., Aonuma, K., and Marumo, F. (1995). Differential localization of atrial natriuretic peptide and skeletal alpha-actin messenger RNAs in left ventricular myocytes of patients with dilated cardiomyopathy. J. Am. Coll. Cardiol. *26*, 85–92.

Trivieri, M.G., Oudit, G.Y., Sah, R., Kerfant, B.G., Sun, H., Gramolini, A.O., Pan, Y., Wickenden, A.D., Croteau, W., Morreale de Escobar, G., et al. (2006). Cardiac-specific elevations in thyroid hormone enhance contractility and prevent pressure overload-induced cardiac dysfunction. Proc. Natl. Acad. Sci. USA *103*, 6043–6048.

Westfall, M.V., Rust, E.M., and Metzger, J.M. (1997). Slow skeletal troponin I gene transfer, expression, and myofilament incorporation enhances adult cardiac myocyte contractile function. Proc. Natl. Acad. Sci. USA *94*, 5444–5449.

Xin, M., Kim, Y., Sutherland, L.B., Murakami, M., Qi, X., McAnally, J., Porrello, E.R., Mahmoud, A.I., Tan, W., Shelton, J.M., et al. (2013). Hippo pathway effector Yap promotes cardiac regeneration. Proc. Natl. Acad. Sci. USA *110*, 13839–13844.

Yang, X., Rodriguez, M., Pabon, L., Fischer, K.A., Reinecke, H., Regnier, M., Sniadecki, N.J., Ruohola-Baker, H., and Murry, C.E. (2014). Tri-iodo-l-thyronine promotes the maturation of human cardiomyocytes-derived from induced pluripotent stem cells. J. Mol. Cell. Cardiol. *72*, 296–304.

Zhang, J., Klos, M., Wilson, G.F., Herman, A.M., Lian, X., Raval, K.K., Barron, M.R., Hou, L., Soerens, A.G., Yu, J., et al. (2012). Extracellular matrix promotes highly efficient cardiac differentiation of human pluripotent stem cells: the matrix sandwich method. Circ. Res. *111*, 1125–1136.

Stem Cell Reports, Volume *3*
Supplemental Information

Acquisition of a Quantitative, Stoichiometrically Conserved Ratiometric Marker of Maturation Status in Stem Cell-Derived Cardiac Myocytes

Fikru B. Bedada, Sunny S-K. Chan, Stefania K. Metzger, Liyang Zhang, Jianyi Zhang, Daniel J. Garry, Timothy J. Kamp, Michael Kyba, and Joseph M. Metzger

Figure S1 related to Figure 1 and 2

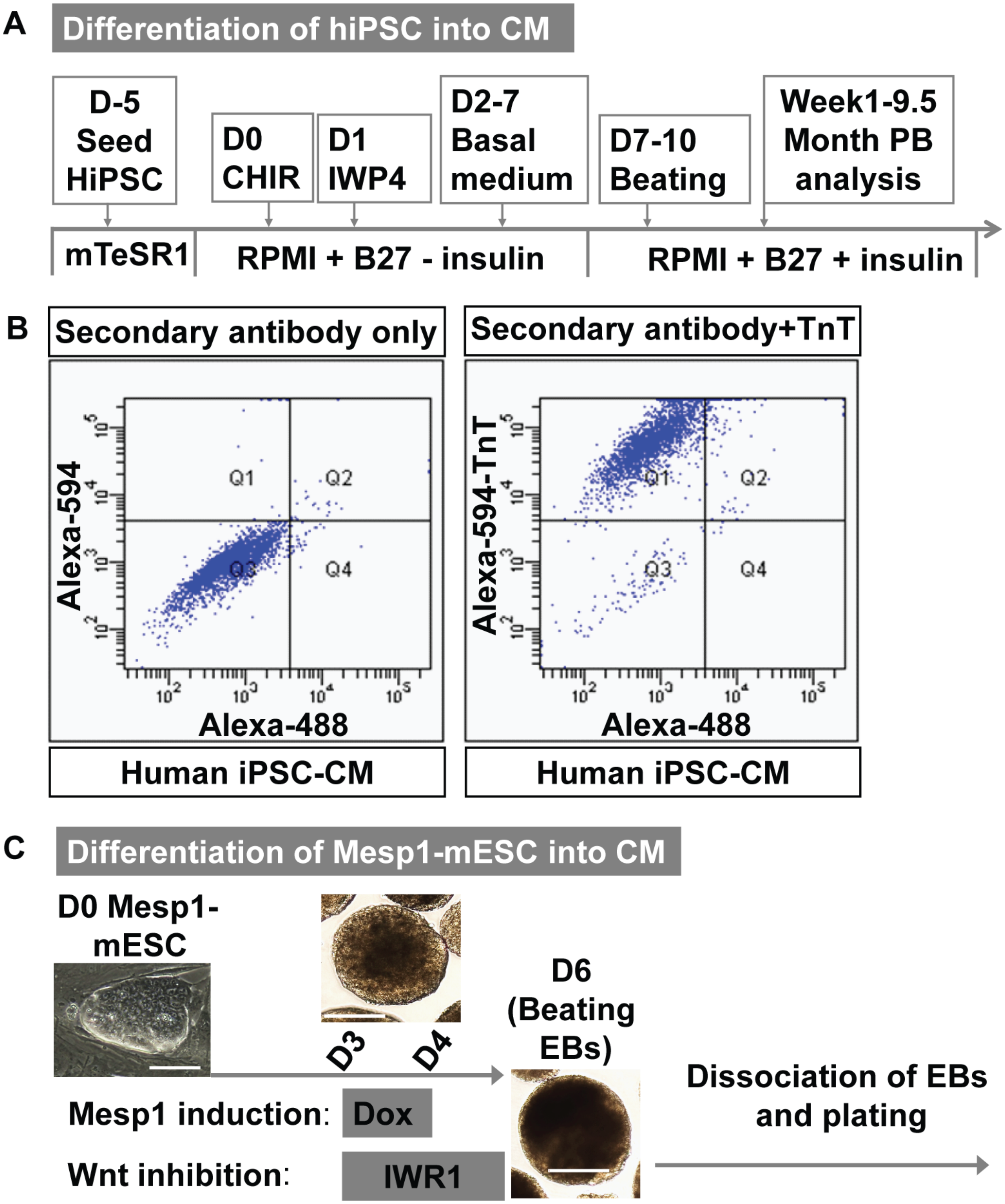

Figure S2 related to figure 1

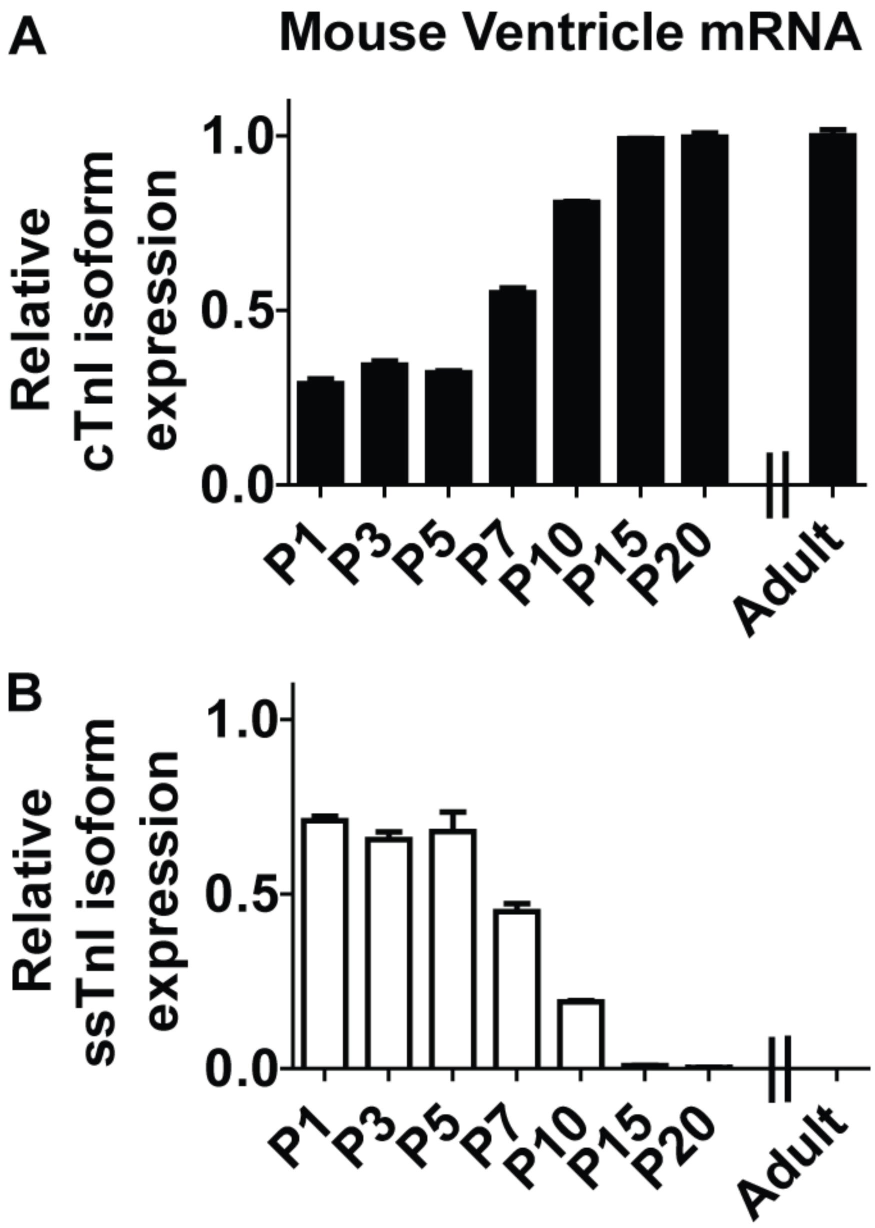

Figure S3 related to figure 1 and 2

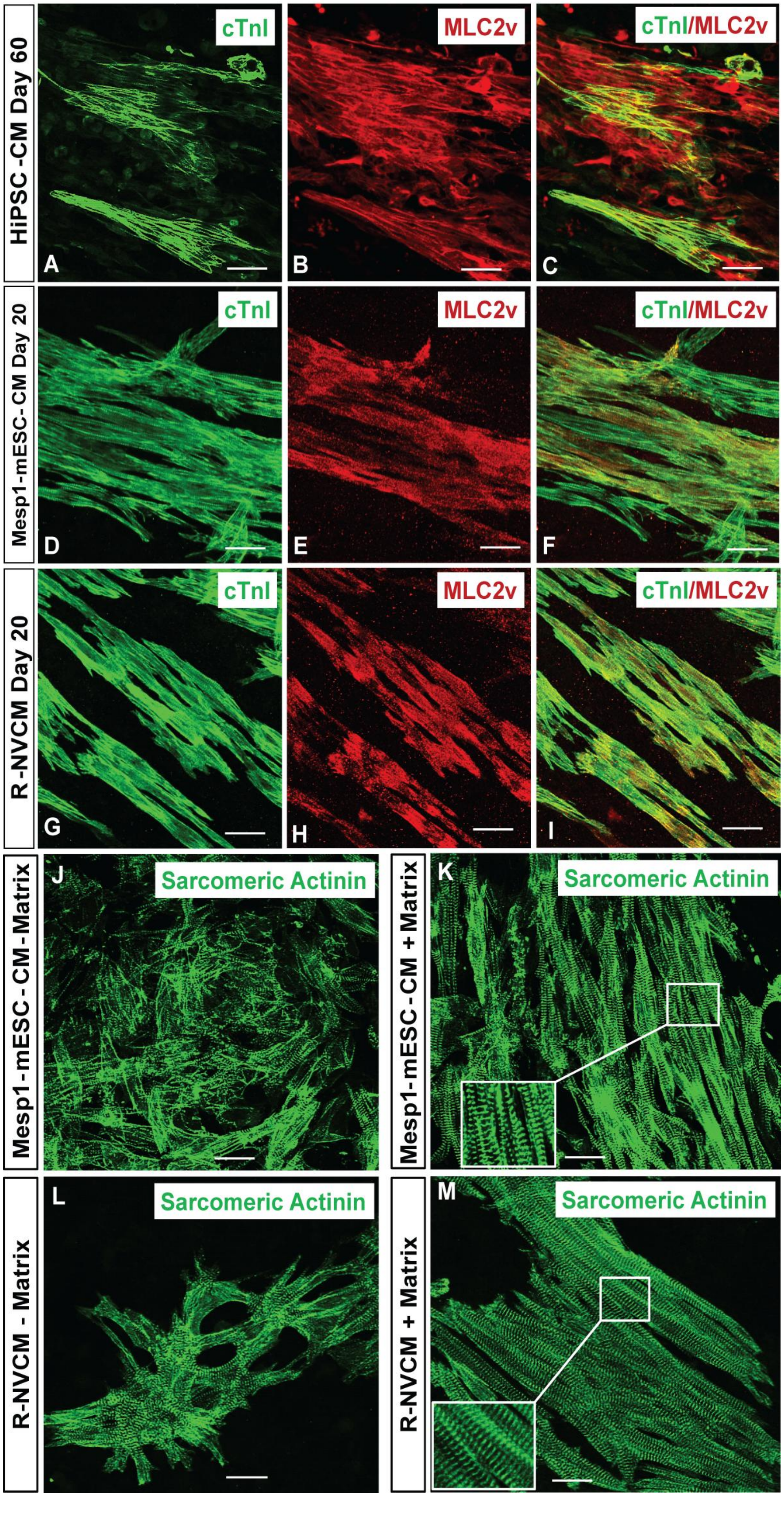

Figure S4 related to figure 2

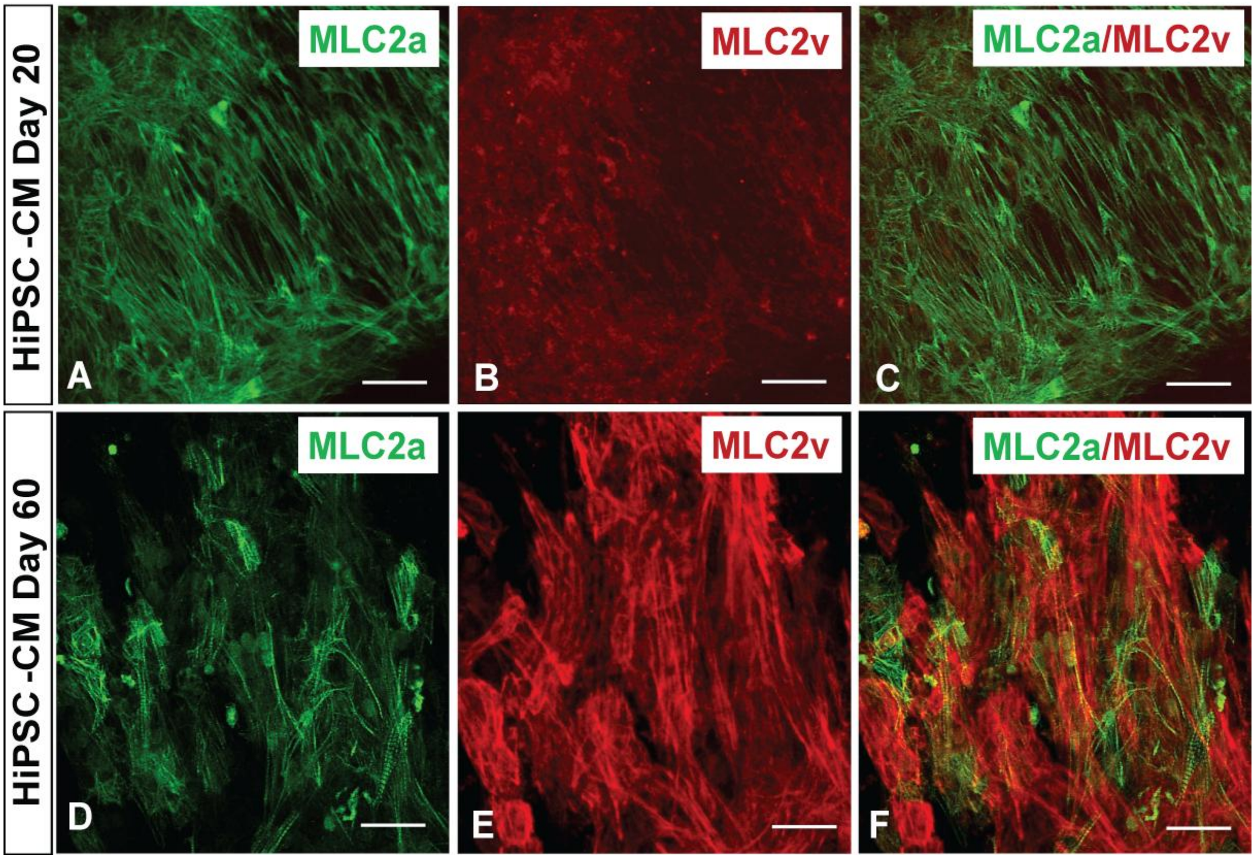

Figure S5 related to figure 5

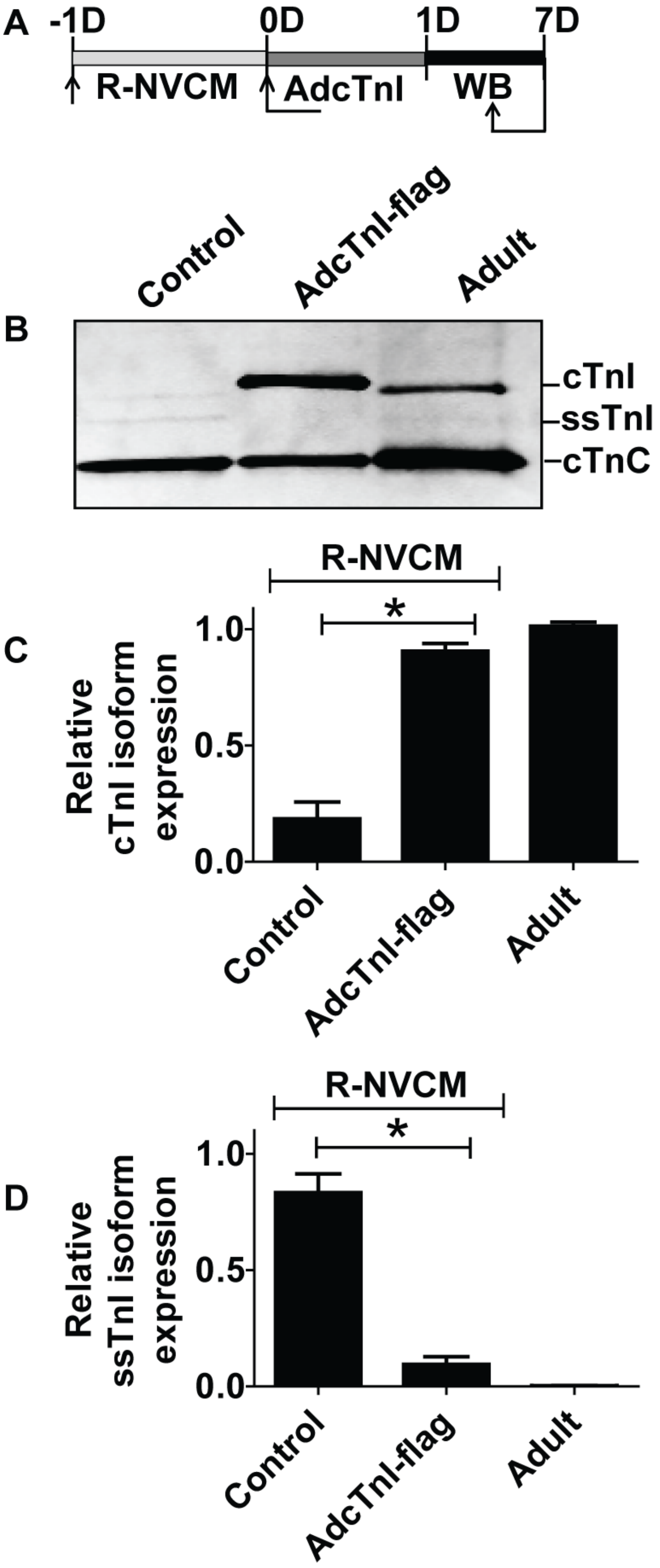

Supplemental Figure Legends

Figure S1 related to figure 1 and 2. Differentiation protocol for hiPSC and Mesp1-mESC-CMs. (A) Schematic of the differentiation protocol for hiPSC-CMs as described in the material and methods. (B) FACS analysis of TnT expression and (C) schematic of differentiation protocol for Mesp1-mESC-CMs as described in the material and methods. The scale bar is 50 µm for undifferentiated mESC and 200 µm for day 4 and day 6 EBs.

Figure S2 related to figure 1. **Developmental expression of *TNNI* isoform mRNAs in ventricles of mouse pups in vivo.** The temporal developmental transition of ssTnI to cTnI was analyzed at the mRNA level to evaluate the transcriptional regulation in vivo. To this effect, RNA samples were obtained from ventricles of postnatal mouse pups at different time points including P1-P20. Ventricles from an adult mouse used as a control. Quantitative RT-PCR data shows gradual increase in the level of cTnI and concomitant reduction in ssTnI for mouse pups in vivo. Data analyzed by two-way ANOVA with Bonferroni post-hoc test and expressed as mean ± SEM, n=3 independent experiments, $P < 0.05$.

Figure S3 related to figure 1 and 2. Sarcomeric alignment and co-localization of mature and chamber specific markers in hiPSC-CMs and rodent-CMs. Immunohistochemistry was used to evaluate the localization of cTnI (mature marker) and MLC2v (ventricular marker) in 60 days post spontaneous beating hiPSC-CM (top panels A-C); Mesp1-mESC-CMs (middle panels D-F) and R-NVCMs (lower panels G-I). The images were taken with 40x objective; calibration bar is 50 µm. Rodent-CMs repopulating the biological matrix aligned and displayed distinct sarcomere organization within 7-15 days of culture with extensive synchronized spontaneous contraction compared with those cultured in the absence of biological matrix. Mesp1-mESC-CM cultured in the absence of the biological matrix did not align well in culture (Panel J left) whereas Mesp1-mESC-CM repopulating the biological matrix aligned and displayed marked sarcomere organization (Panel K right). R-NVCM cultured in the absence of the biological matrix did not align well (Panel L left) whereas R-NVCM repopulating the biological matrix aligned and displayed marked sarcomere organization (Panel M right). The images were taken with 40x objective; calibration bar is 50 µm.

Figure S4 related to figure 2. Acquisition of chamber specific markers in hiPSC-CMs. HiPSC-CMs were stained with MLC2a (green) and MLC2v (red) specific antibodies. Representative images are shown for day 20 MLC2a (A), MLC2v (B) and merged images (C). Day 60 MLC2a is shown in (D), MLC2v (E) and merged images (F). The images were taken with 40x objective; calibration bar is 50 µm.

Figure S5 related to figure 5. Acquisition of the mature signature by stoichiometric gene replacement in rodent-CM. Schematic of AdcTnI (flag tagged) gene transduction in day 1 R-NVCM (A). Western blot of AdcTnI(flag tagged) treated R-NVCM is shown in (B). The direct effect of AdcTnI(flag tagged) on acquisition of cTnI is quantified in (C) and suppression of ssTnI is quantified in (D).). cTnC is used as cardiac specific loading control and for normalization/quantification. Data analyzed by One-way ANOVA with Bonferroni post-hoc test and expressed as mean ± SEM, n=3 independent experiments, ∗=P <0.05.

Supplemental Experimental Procedure

Preparation of thin slices of biological matrix for culture of hiPSC-CM and rodent-CM

Whole hearts were isolated from female Sprague–Dawley rats following anesthetization by isoflurane inhalation. Freshly isolated rat hearts were placed in 1 mm stainless steel rat heart slicer block with slice intervals spaced 1.0mm apart (Zivic Instruments). The 1.0 mm transverse section slice intervals allow slices of repeatable and reproducible uniformly sized sections. The 1 mm thin heart sections were placed in 1% SDS with continuous agitation and regular change of the detergent for 72 hrs. The matrix was washed and rinsed continuously with PBS for 2-3 days and kept at 4°c in PBS until usage.

Next, $18mm^2$ coverslips were placed in 6 well plates and the surface aseptically coated with 0.1mg/ml poly-D-lysine (Sigma) for 5 min and then rinsed with tissue culture grade water and allowed to dry for 2hrs. The thin slice of decellularized matrix was placed on poly-D-lysine-coated coverslips for 15-30min. Following this, hiPSC-CMs or rodent-CMs were placed in the decellularized cardiac thin sections for 1-2 hrs and medium changed every 2-3 days thereafter. As a control, hiPSC-CM or rodent-CM are cultured in coverslips coated with matrigel only.

Flow Cytometry Analysis

Dissociated single hiPSC-CMs were fixed with 2% (vol/vol) paraformaldehyde for 30min at room temperature and stained with TnT primary antibody for 1hr at room temperature and Alexa 594 secondary antibodies for 1hr at room temperature in PBS plus 0.01% (vol/vol) Triton X-100 and 1% (wt/vol) BSA. HiPSC-CMs that were stained with secondary antibody only were used as control for FACS gating. The expression was analyzed using BD FACSAriaII (BD Biosciences, San Diego, CA, USA).

Quantitative RT-PCR

Total RNA was isolated from hiPSC-CM, rodents-CMs using Trizol reagent (Invitrogen) following manufacturer's instructions. In the reverse transcription (RT) step, cDNA was reverse transcribed from 500 ng total RNA samples using TaqMan Reverse Transcription Kit. PCR products were amplified from cDNA samples using the SYBR Green PCR Master Mix (Invitrogen). All qPCR assays were performed in duplicate on all control and experimental group and normalized against control myocytes. Relative quantitation analysis of gene expression was conducted according to the $2^{-\Delta\Delta CT}$ method as described [1]. cTnC was used as endogenous internal standard for cTnI:ssTnI expression analysis to determine the abundance of amplified target gene within the same sample.

Supplemental References

1. Livak, K.J. and T.D. Schmittgen, *Analysis of relative gene expression data using real-time quantitative PCR and the 2(-Delta Delta C(T)) Method.* Methods, 2001. **25**(4): p. 402-8.
